# Structural and chemical evolution in layered oxide cathodes of lithium-ion batteries revealed by synchrotron techniques

**DOI:** 10.1093/nsr/nwab146

**Published:** 2021-08-17

**Authors:** Guannan Qian, Junyang Wang, Hong Li, Zi-Feng Ma, Piero Pianetta, Linsen Li, Xiqian Yu, Yijin Liu

**Affiliations:** Stanford Synchrotron Radiation Lightsource, SLAC National Accelerator Laboratory, Menlo Park, CA 94025, USA; Department of Chemical Engineering, Shanghai Electrochemical Energy Device Research Center (SEED), School of Chemistry and Chemical Engineering, Frontiers Science Center for Transformative Molecules, Shanghai Jiao Tong University, Shanghai 200240, China; Stanford Synchrotron Radiation Lightsource, SLAC National Accelerator Laboratory, Menlo Park, CA 94025, USA; Beijing Advanced Innovation Center for Materials Genome Engineering, Key Laboratory for Renewable Energy, Beijing Key Laboratory for New Energy Materials and Devices, Institute of Physics, Chinese Academy of Sciences, Beijing 100190, China; Beijing Advanced Innovation Center for Materials Genome Engineering, Key Laboratory for Renewable Energy, Beijing Key Laboratory for New Energy Materials and Devices, Institute of Physics, Chinese Academy of Sciences, Beijing 100190, China; Department of Chemical Engineering, Shanghai Electrochemical Energy Device Research Center (SEED), School of Chemistry and Chemical Engineering, Frontiers Science Center for Transformative Molecules, Shanghai Jiao Tong University, Shanghai 200240, China; Stanford Synchrotron Radiation Lightsource, SLAC National Accelerator Laboratory, Menlo Park, CA 94025, USA; Department of Chemical Engineering, Shanghai Electrochemical Energy Device Research Center (SEED), School of Chemistry and Chemical Engineering, Frontiers Science Center for Transformative Molecules, Shanghai Jiao Tong University, Shanghai 200240, China; Shanghai Jiao Tong University Sichuan Research Institute, Chengdu 610213, China; Beijing Advanced Innovation Center for Materials Genome Engineering, Key Laboratory for Renewable Energy, Beijing Key Laboratory for New Energy Materials and Devices, Institute of Physics, Chinese Academy of Sciences, Beijing 100190, China; Stanford Synchrotron Radiation Lightsource, SLAC National Accelerator Laboratory, Menlo Park, CA 94025, USA

**Keywords:** layered battery cathode, synchrotron, electro-chemo-mechanical interplay, surface, bulk

## Abstract

Rechargeable battery technologies have revolutionized electronics, transportation and grid energy storage. Many materials are being researched for battery applications, with layered transition metal oxides (LTMO) the dominating cathode candidate with remarkable electrochemical performance. Yet, daunting challenges persist in the quest for further battery developments targeting lower cost, longer lifespan, improved energy density and enhanced safety. This is, in part, because of the intrinsic complexity of real-world batteries, featuring sophisticated interplay among microstructural, compositional and chemical heterogeneities, which has motivated tremendous research efforts using state-of-the-art analytical techniques. In this research field, synchrotron techniques have been identified as a suite of effective methods for advanced battery characterization in a non-destructive manner with sensitivities to the lattice, electronic and morphological structures. This article provides a holistic overview of cutting-edge developments in synchrotron-based research on LTMO battery cathode materials. We discuss the complexity and evolution of LTMO’s material properties upon battery operation and review recent synchrotron-based research works that address the frontier challenges and provide novel insights in this field. Finally, we formulate a perspective on future directions of synchrotron-based battery research, involving next-generation X-ray facilities and advanced computational developments.

## Teaser

This article reviews novel synchrotron techniques for studying the structural, chemical, morphological and dynamic complexities in state-of-the-art industry-relevant lithium-ion battery cathode materials.

## INTRODUCTION

### Layered transition metal oxide battery cathode

With a broad range of applications in electric vehicles (EV), grid storage and consumer electronics, the lithium-ion battery (LIB) has become a major player in global energy storage solutions in recent years [1–3]. It is predicted that, by 2025, the global lithium-ion battery market will rise to nearly $100 billion and the shipment volume will reach nearly 440 GWh [4]. The rapid growth of the LIB portfolio is attributed to its favorable characteristics including high energy density, high power density and environmental friendliness, which also set the targets for further research efforts in this field. Although all the battery components are important to the ultimate performance and are subject to intense research, the LIB cathode remains the most significant bottleneck for further improving the energy density. Among the materials that have been considered and researched as candidates for the battery cathode, layered transition metal oxides (LTMO) [5,6] are the most auspicious. Examples of successfully commercialized LTMO battery cathodes include lithium cobalt oxide (LCO) [7–9] for powering consumer electronics and lithium nickel cobalt manganese oxide (NMC) [10–12] in EV applications, demonstrating a tremendous market value.

**Figure 1.**
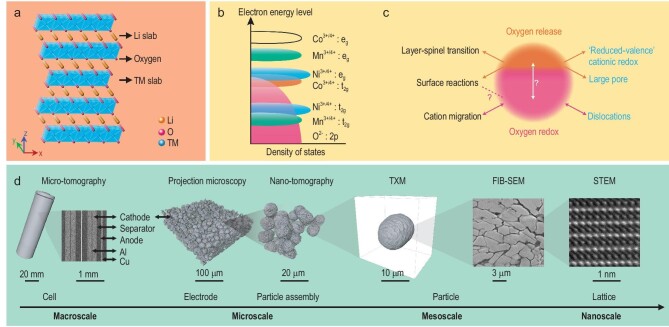
Overview of the lattice structure, electronic structure and micro-morphology of the LTMO cathode. (a) Crystal model of layered LTMO (TM: 3d transition metal cations, Ni, Co, or Mn). (b) Schematic illustration of the electronic structures of the key elements in LTMO. (c) Schematic illustration of the oxygen evolution and the associated degradation mechanisms to the LTMO cathode. Adapted with permission from [18], Copyright 2018, Springer Nature. (d) Multi-scale and high dimensional characterization for real-world LTMO-based LIB.

The LTMO features a layered lattice structure that is essential to the reversible lithium (de)intercalation. As illustrated in Fig. 1a, LTMO exhibits an *α*-NaFeO_2_ structure with *R-3m* space group, in which lithium ions and transition metal cations occupy octahedral 3a and 3b sites, respectively, while oxygen anions occupy the octahedral 6c sites with cubic close packing [13]. In the layered LTMO lattice, the diffusion of lithium ions strongly favors an in-plane, two-dimensional geometry and is mediated by a divacancy mechanism, in which one lithium ion hops to a vacant site that is part of divacancies through a tetrahedral site [14,15]. Upon battery charging, extraction of lithium ions from the host LTMO matrix is accompanied by redox reactions of transition metal cations and, potentially, oxygen anions. The LTMO’s lattice structural stability is closely associated with these intertwined redox centers [11,16]. We show, in Fig. 1b, the electronic structure of four representative elements in LTMO compounds: nickel, cobalt, manganese and oxygen. The overlap of the Co^3+^/Co^4+^*t*_2g_ band with the top of the O^2–^ 2*p* band indicates that the empty states undergo a strong anion-*p*/M*-d* covalent admixture. For example, the O^2–^ 2*p* molecular orbitals tend to trap itinerant holes when the LCO is charged above 4.5 V, leading to O3 → H1-3 phase transitions [17]. The Ni^3+^/Ni^4+^*e*_g_ band has a smaller overlap with the O^2–^ 2*p* band. As a consequence, the Ni^4+^ state can be partially reached by extracting more lithium ions without severely jeopardizing the lattice structural stability. Unlike Ni and Co, the Mn^3+^/Mn^4+^*e*_g_ band has no overlap with the O^2–^ 2*p* band, resulting in a superior structural robustness. The chemical evolution of oxygen anions in LTMO can have a more profound impact beyond deteriorating the LTMO’s structural stability through oxygen release and the associated multi-scale structural degradation. Recently, the oxygen redox mechanism in LTMO has drawn a lot of attention (see illustration in Fig. 1c) because, if this could be made controllable and reversible, it is hoped it could support higher capacity and power [18,19]. In LTMO with multiple co-existing transition metal cations, the interplay among all the different redox centers could distort their respective electronic structures in a complex, interdependent, and dynamic manner, formulating a highly sophisticated electrochemical reaction upon battery cycling.

The strong desire to further advance the energy/power density and the stability/safety has motivated development of several new types of LTMO [6,20], featuring an even higher degree of hierarchical and high-dimensional complexity in structure, morphology, composition and local chemistry. For example, compositional gradient [21,22] and ordered primary particle packing [23,24] are found to be effective for improving the cycling stability. The lattice defect engineering and grain boundary modification, on the other hand, could improve the energy density by promoting stability at deeply charged states [25–27]. Engineering of particle formation, for example single-crystallization [28–30], is another active research direction. To truly unleash the potential of these new LTMO materials, rational microstructure design at the electrode level is indispensable. In Fig. 1d, we illustrate the multi-scale morphological complexity in a real-life LTMO cathode from the cell level down to the atomic scale. A practical LTMO composite cathode accentuates distinct surface and bulk reactions that are fundamental to the battery performance. An in-depth and comprehensive understanding of the depth-dependent structural and chemical interplay in LTMO could critically inform research efforts in this field. It not only exhibits tremendous scientific significance, but also is closely relevant to the battery industry. With the enormous amount of effort being devoted to this research direction, here we present a high-level overview of this field with an emphasis on the valuable insights offered by advanced synchrotron characterization techniques.

### Synchrotron characterization techniques

**Figure 2.**
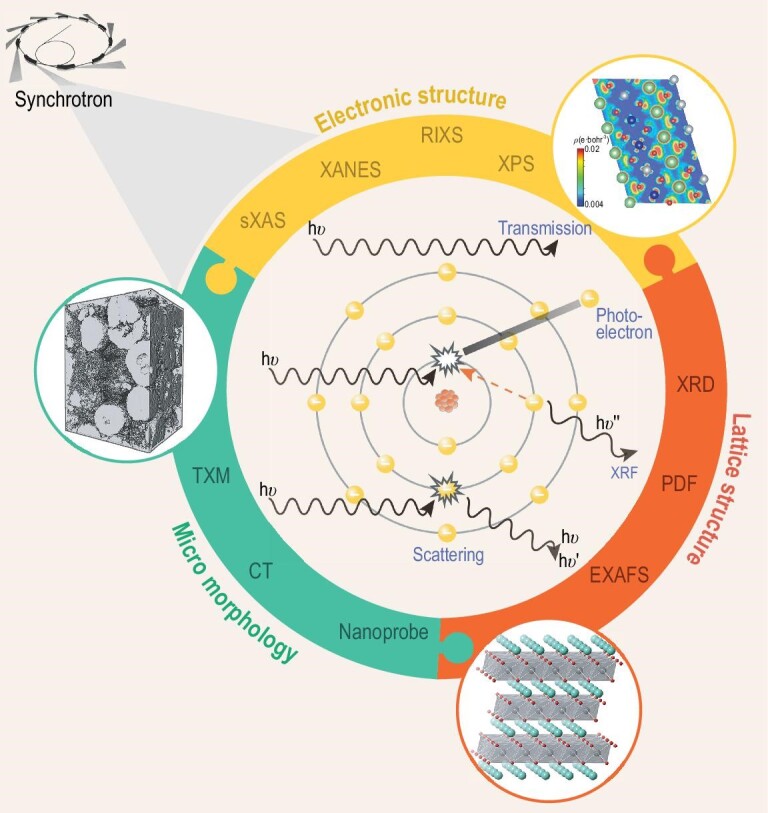
Schematic illustration of the interaction between the synchrotron X-rays and the material. A number of synchrotron characterization techniques are listed, highlighting their respective sensitivity to the lattice structure, the electronic structure and the micro-morphology. Adapted with permission from [32], Copyright 2017, IOP.

A systematic investigation of the fundamental yet complicated reaction mechanisms in LTMO relies on advanced characterization techniques. In this respect, synchrotron X-ray techniques have demonstrated unique advantages with excellent multi-scale resolution and multi-modal sensitivity. The principle of synchrotron radiation is based on classical electrodynamics: when a charged particle traveling at a speed close to the speed of light is forced to change its moving direction, electromagnetic radiation will be emitted [31]. When X-rays are delivered to the sample, they interact with the matter in a few different ways at different likelihood, leading to several signals that are associated with different material properties, respectively. Generally speaking, absorption, phase-shifting and scattering are three different basic interactions between the X-rays and the matter, which derive various X-ray characterization techniques that are sensitive to the lattice structure, electronics structure [32] and the micro-morphology (see illustration in Fig. 2). A more detailed introduction to these techniques is available in the Supplementary data, section I and the following sections.

We acknowledge that there are several high-quality reviews [31,33–35] in the literature, featuring battery materials research using synchrotron X-ray techniques. These articles often highlight the characterization techniques with a broad range of case studies covering many different battery materials and components. They not only highlight the tremendous research efforts in this field, but also reflect the enormous complexities in both the technical and the material perspectives. Considering the critical role of LTMO in today's lithium-ion batteries and the enormous research interests from both industry and academia, here we formulate a critical review of recent advances in research into LTMO using synchrotron X-ray techniques. More specifically, in this review, we choose to focus on discussing the Ni/Co/Mn-based LTMO, which dominates today's market while showing prominent potential as a next-generation battery cathode candidate. The synchrotron techniques reviewed here are broadly applicable to the other relevant material systems and the broader impact of these synchrotron techniques for energy material research can be extrapolated from our review. In this article, the application of synchrotron techniques in studying LTMO battery cathode materials will be reviewed from three different perspectives: the lattice structures, electronic structures and multi-scale morphology, as illustrated in Figs 1 and 2. We will systematically discuss the relevant techniques and the scientific implications will be summarized with a focused theme on the depth-dependent structural and chemical interplay and evolution in LTMO upon battery cycling. Finally, we will conclude our review by discussing the future perspectives, including the data science approaches, the projected major synchrotron and X-ray free electron laser upgrades, and their impact on the research for next-generation high energy and power density battery materials.

## SYNCHROTRON CHARACTERIZATION OF LTMO’S LATTICE STRUCTURAL COMPLEXITY AND EVOLUTION

In an ideal electrochemical process, the lattice structure of LTMO would periodically contract and expand as the lithium-ions reversibly intercalate and deintercalate. In practice, however, the reversibility of lattice structure can be affected by a number of factors, including their intrinsic properties, the delithiation amount, the charging/discharging protocols, etc. While some detailed descriptions of the LTMO’s lattice structural complexity can be found in the Supplementary data, section II, here we discuss a few recent examples of synchrotron characterization of LTMO’s lattice structures.

**Figure 3.**
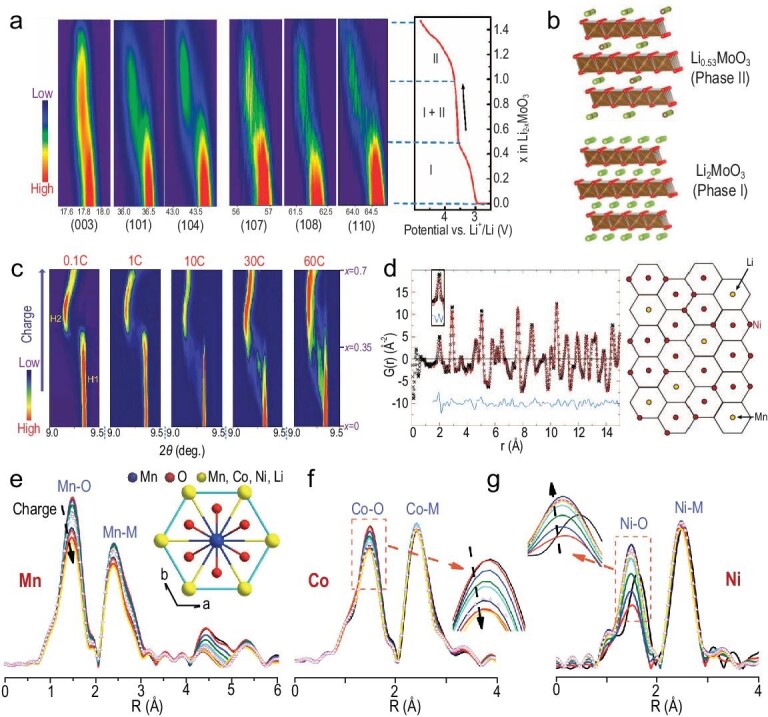
Synchrotron-based structural studies of LTMO cathode materials. (a) *In situ* XRD pattern and charge curve of Li_2−x_MoO_3_ cathode during delithiation. Adapted with permission from [37], Copyright 2014, Springer Nature. (b) Structure models for two phases formed during the charge process. Adapted with permission from [37], Copyright 2014, Springer Nature. (c) *In situ* XRD of LiNi_1/3_Mn_1/3_Co_1/3_O_2_ during the first charge at different C rates (0.1, 1, 10, 30 and 60C). Adapted with permission from [38], Copyright 2016, WILEY‐VCH. (d) Pair distribution function *G*(*r*) of LiNi_0.5_Mn_0.5_O_2_ obtained from synchrotron X-ray total scattering and in-plane cation ordering of LiNi_0.5_Mn_0.5_O_2_ derived from reverse Monte-Carlo (RMC) refinement. Adapted with permission from [40], Copyright 2005, American Chemical Society. (e–g) Fourier transformed EXAFS Mn, Co and Ni *K*‐edge spectra of Li_1.2_Ni_0.15_Co_0.1_Mn_0.55_O_2_ collected during the initial charge process. Adapted with permission from [45], Copyright 2013, WILEY‐VCH.

The lattice structures of cathode materials are intimately correlated with the battery performance metrics, for example power and energy density, cyclic span and safety [36]. Thus, an in-depth understanding of the structure–performance relationship is pivotal to optimization of the current cathode material systems as well as to developing new ones with superior electrochemical properties. This necessitates the study of structural evolution as the system undergoes the electrochemical reactions, including changes in the lattice parameters, phase transformation and atomic occupancy. *Insitu* X-ray diffraction (XRD) is a widely used method for probing the crystal structure and phase transformation for crystalline electrode materials during cycling. For instance, Zhou *et al*. utilized *insitu* XRD to investigate the structural evolution of the Li_2_MoO_3_ cathode during the charge–discharge process and found an ‘abnormal’ unit cell breathing mechanism for the cathode materials [37]. As presented in Fig. 3a, the XRD peaks of pristine and charged Li_2_MoO_3_ can be indexed according to a layered structure with the *R-3m* space group. The *insitu* XRD data of the Li_2−x_MoO_3_ electrode upon battery charging demonstrated a solid solution reaction in the region of 0 < *x *< 0.5. All the major X-ray reflections are found to shift toward the lower angle side, indicating a larger *d*-spacing of phase I. Subsequently, a new phase (phase II), which shares a similar layered structure with phase I but with larger lattice parameters, emerges in the region of 0.5 < *x *< 1, suggesting the occurrence of a two-phase reaction. With delithiation further proceeds over *x* >1, a solid solution reaction of phase II occurred, while phase I disappeared. Interestingly, while the widely used layered LiMO_2_ (M = Co, Ni, Mn) shows shrinkage in the lattice parameters of *a* and *b* during charging, the Li_2_MoO_3_ exhibits a reversed trend in the lattice parameter's evolution. Such ‘abnormal’ behavior can be explained by the specific attributes of metal–metal bonding in the materials that control the *a(b)* evolution during charging.

When it comes to the design of battery materials for high power applications, it is crucial to establish a fundamental understanding of the structure evolution that happens in the electrode materials during the fast charge–discharge process, which could be quite different from the case at small C-rates. Thus, time-resolved X-ray diffraction (TR-XRD) emerges as a powerful technique for investigating the fast dynamic properties of electrode materials at high current densities. Taking advantage of the fast data acquisition capability and high brightness of synchrotron X-ray, Zhou *et al.* conducted *insitu* synchrotron TR-XRD to study the phase transition process of layered LiNi_1/3_Mn_1/3_Co_1/3_O_2_ under different cycling rates [38]. As shown in Fig. 3c, both the charging processes of the material under the current rates of 0.1 and 1 C show two different regions of solid solution reactions involving hexagonal phases of H1 and H2, between which there is another region with the coexisting H1 and H2 phases. Importantly, with the charging rate further increased to 10 C, a new intermediate phase emerged between the two solid solution reaction regions, while the peaks of this structure become increasingly pronounced at even higher rates of 30 C and 60 C. Besides, an abnormal Li-poor region with tetrahedral Li occupation and a normal Li-rich region with octahedral Li occupation were visualized in the partially charged sample with scanning transmission electron microscopy (STEM). This suggests that the intermediate phase can serve as a buffer zone between the Li-rich and Li-poor phases, which could reduce the local stress and strain that are induced by the charge heterogeneity and, therefore, stabilize the material structure.

Although XRD techniques are useful for structural analysis of crystalline materials, they provide limited structural information for samples without long-range order, such as amorphous materials and materials in the form of nano-sized crystals. For example, it is challenging to utilize XRD for probing the reaction mechanism of conversion-type electrode materials because of the nature of highly disordered nano particles formed during the conversion reaction process. To resolve this issue, the pair distribution function (PDF) has been used for battery research, complementing the XRD approaches. While XRD concerns only Bragg scattering and probes’ averaged long-range structural features, the PDF technique can investigate the local structure of the materials with short-range ordering using the total scattering signal, including both Bragg and diffuse scattering [39]. Specifically, this can provide local information regarding atomic pair distribution, which is closely related to the electrochemically driven chemical, structural and morphological transformations.

A typical example to explain how PDF reveals the short-range ordering in LiNi_0.5_Mn_0.__5_O_2_ material is provided by Breger *et al.* (Fig. 3d). They reported that there is a strong site occupation correlation between Ni and Mn cations in the first and second coordination shells, which results in non-random distribution in the short-range scale [40]. A reverse Monte-Carlo (RMC) simulation of the PDF data was conducted using a ‘big box’ structure model, which contains hundreds to thousands of atoms. In RMC simulation, one can build a three-dimensional non-periodic atomic structure to quantify the local ordering over a longer distance [41,42]. This approach offers sensitivity to the short-to-mid-range ordered local coordination environments that could be overlooked in conventional crystallography. Through this approach, several hidden structural features of short-range ordering in the material are revealed. One is that the Ni cations are likely surrounded by Mn and Li cations in the first and second coordination shells, respectively. Another structural characteristic is that about 10% of the Li/Ni mixing exists in the structure, meaning that about 10% of the Li ions from the Li layer exchange with the Ni cations in the TM layers. Moreover, the Li ions in the transition metal layer are more likely to be surrounded by Mn cations rather than by Ni cations in their first coordination shell. These local structural features cannot be deduced from fitting the conventional XRD patterns, which are associated with the averaged bulk structure of the material. Therefore, combining PDF data with advanced fitting/simulation methods can be an effective method to unlock the hidden structural information for battery materials.

In addition to the PDF analysis, X‐ray absorption fine structure spectra (XAFS) offers another powerful technique for studying the local structure around active atoms at atomic and molecular scales. Based on the energy range, an XAFS spectrum can be separated into two regions, X-ray absorption near edge structure (XANES) and extended X-ray absorption fine structure (EXAFS). While the XANES spectra are sensitive to the electronic structure and local chemical environment, the EXAFS spectra are often used to determine the local structural information, including bonding distances, coordination number, neighboring atom species, lattice distortion and phase transformation. As the local lattice structure is important for modulating the ionic diffusion, phase composition and electrochemical reaction mechanisms, probing the local structural properties is crucial to constructing a full view of the material structure and to enabling a rational material design for improved electrochemical performance.

EXAFS analysis has also been utilized to evaluate the cycling-induced change of crystal structures and structural instability of LTMO cathode, which is closely related to the capacity degradation mechanism of LTMO. For example, Kim and Yo used EXAFS to investigate the interatomic distance and structure distortions of LiCo_0.85_Al_0.__15_O_2_ during battery charging [43]. As elucidated by the *k*‐space and *R*‐space spectra from EXAFS analysis, all bond pairs exhibit a decrease in interatomic distance and the crystal structure becomes more distorted as evidenced by the increased Debye-Waller factor. Thus, a change of local structures of the cathode material can be a major reason for the capacity degradation. Separately, Tsai *et al.* conducted *insitu* XAFS to study structural evolution in the NMC cathode upon cycling [44]. The *insitu* XAFS data confirmed that the charge compensation mechanism of the NMC cathode is mainly contributed by Ni and O, as demonstrated by the change of Ni−O bond length with Ni^2^^+^ oxidization. While the structural distortion is revealed by the change of Debye-Waller factor as the Ni^3^^+^ is the JT effect active species, which can cause a change in the bond length during the charge and discharge process. Further, Yu *et al.* applied *insitu* XANES and EXAFS to study the local structure changes around Ni, Mn and Co in Li‐rich layered Li_1.2_Ni_0.15_Mn_0.55_Co_0.1_O_2_ during the first charge [45]. Although the edge energy of the Mn and Co *K*‐edge spectra maintains the same position during the first delithiation, the shape of the XANES spectra and the Fourier transformed (FT) EXAFS spectra demonstrate significant changes in the peak intensity of Co‐O and Mn−O bonds. This phenomenon suggests that Mn and Co are responsible for the charge compensation process for this electrode material during delithiation, and have varying tendencies for the process in different states of charge (Fig. 3e–g) [45]. Based on the EXAFS analysis, it was proposed that Co and Mn are mainly related to the voltage slope and voltage plateau regions, respectively. Further analyzing the Debye-Waller factors, changes in the local environment around Mn also suggest Mn-related structural distortion behavior, which could be an important reason for the capacity decay over prolonged cycling. Thus, *insitu* EXAFS has been used for elucidating the local structure evolution and charge compensation mechanism for Li‐rich layered Li_1.2_Ni_0.15_Co_0.1_Mn_0.55_O_2_, which is different from that of conventional NMC.

Taken together, these studies on the electrochemical behavior of various cathode materials have constructed a comprehensive understanding of the electrochemistry for the LIBs, which is of significant value to the design, synthesis and development of advanced cathode materials in the future.

## SYNCHROTRON CHARACTERIZATION OF LTMO’S ELECTRONIC STRUCTURAL COMPLEXITY AND EVOLUTION

The electronic structure of transition metal cations and oxygen anions in LTMO plays a pivotal role in the battery's functionality. We demonstrate, in detail, the electronic structural complexity of LTMO in real-world operating batteries in the Supplementary data, section III. Synchrotron-based X-ray spectroscopy techniques have distinct advantages in revealing the evolution of LTMO’s electronic structures effectively and efficiently. Here, we discuss the application of three popular X-ray spectroscopy tools (namely X-ray photoelectron spectroscopy (XPS), X-ray absorption spectroscopy (XAS), and resonant inelastic X-ray scattering (RIXS)) for studying LTMO’s electronic structures.

**Figure 4.**
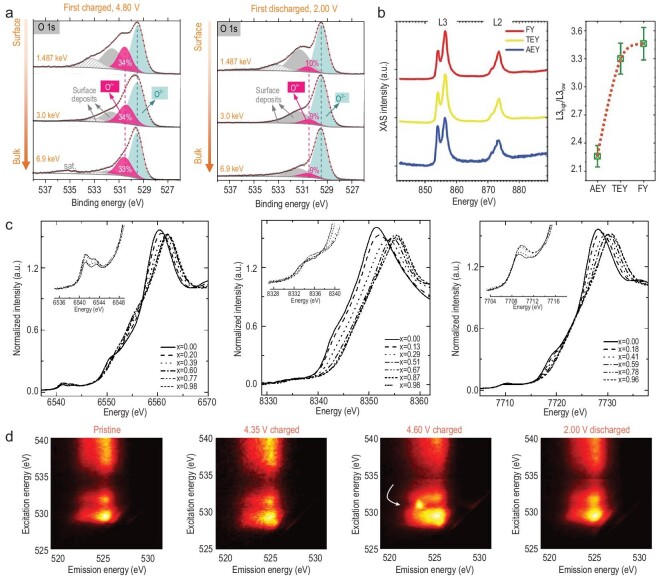
Synchrotron-based X-ray spectroscopic studies of LTMO cathode materials. (a) Depth-dependent XPS signal from Li-rich NMC cathode revealed by HAXPES. Adapted with permission from [48], Copyright 2017, Springer Nature. (b) Soft X-ray absorption spectroscopy (sXAS) data on NMC cathode in Auger electron yield (AEY), total electron yield (TEY) and fluorescence yield (FY) modalities. Adapted with permission from [50], Copyright 2014, Royal Society of Chemistry. (c) Hard X-ray XANES data over the *K*-edges of Ni, Mn and Co in an NMC cathode. Adapted with permission from [56], Copyright 2005, American Chemical Society. (d) Soft X-ray RIXS data on a Li-rich NMC cathode at different state-of-charge. Adapted with permission from [67], Copyright 2017, Springer Nature.

Depending on the X-ray energy and the detected signals, several different X-ray spectroscopic techniques have been broadly applied to energy material research. Before going into more detailed discussions, we first clarify that some of these X-ray spectroscopic techniques are not limited to the synchrotron facilities. For example, the tabletop X-ray-based XPS, which detects and resolves the energy of photoelectrons, is widely available for characterizing the chemical states over the material surface. The use of a tabletop X-ray source is associated with a relatively low intensity and a very limited energy tunability (e.g. Al K${\rm{\alpha }}$ radiation of 1.49 keV) [46], which set some practical constraints, for example in probing depth (${\rm{ <\!5 nm}}$ for 1.49 keV) and in chemical sensitivity. When implemented with a synchrotron source, XPS can be carried out at different photon energies, opening up significant scientific opportunities. For example, the probing depths can be effectively adjusted by varying the incident X-ray energy for hard X-ray photoelectron spectroscopy (HAXPES, 13 nm at 3.0 keV, 29 nm at 6.9 keV and 40 nm at 10 keV) [47]. The chemical state can, therefore, be characterized in a depth-dependent manner through this approach. In the study of materials with very fine particle size (∼100 nm or smaller), it can even be considered as a ‘bulk sensitive’ technique. HAXPES has been utilized to study the chemical state, composition and their depth-dependent interplay in LTMO cathode materials. Here we discuss a HAXPES study, by Assat *et al.*, on the charge compensation mechanism for the anion redox in Li-rich NMC cathode [48]. As shown in Fig. 4a, the first charged and discharged Li-rich NMC cathodes were tested using HAXPES with different probing depths. The surface deposits’ contribution to the O 1s photoelectron signal decreases as the incident photon energy increases from ∼1.5 keV to 6.9 keV. The intensity of O^n–^ and its ratio (the percentage of oxidized lattice oxygen) do not change significantly as a function of the depths, suggesting that the O^n–^ exists both on the surface and in the bulk. The charge-compensation mechanism can be speculated based on evolution of the O^n–^ fraction as a function of the state-of-charge. The authors also reported that the variation of the O^n–^ fraction is rather consistent in the first and second cycles, suggesting fairly good reversibility of oxygen redox in the early cycles of this material. The incomplete reduction of oxygen when discharged to 2.0 V hints at its sluggish kinetics and can be associated with the capacity loss in the first cycle. It is suggested that a potentiostatic hold at 2.0 V may be necessary in the discharging process for reducing O^n–^ toward the full extent.

As we discussed in the previous section, when an X-ray photon is absorbed by the material, a core-level electron is excited to the unoccupied states above the vacuum or Fermi level. When the energy of the photon resonates with the ionization potential of a certain quantum state, the absorption cross-section is maximized and the resulting discontinuity in the absorption spectrum is referred to as an ‘absorption edge’. The energy shift of the spectrum often reflects a change in oxidation state, while the spectrum shape is related to the local chemical environment and the ligand geometry. Therefore, the X-ray absorption spectrum is often used to fingerprint different chemical species. Table S2 lists the absorption edges and their corresponding energies for the primary elements in LTMO material [49]. Here we discuss a few different spectroscopy techniques, including soft X-ray absorption spectroscopy (sXAS), XANES and RIXS, all of which have demonstrated valuable contributions to understanding of the electronic structures in LTMO materials.

Most of the sXAS beamlines can cover the transition metal *L*-edges and O *K*-edge and can probe the valance band of TM 3d and O 2p states. In a sXAS scan, different signals can be detected, formulating different operation modes include Auger electron yield (AEY), total electron yield (TEY) and fluorescence yield (FY). The probing depth of these signals varies, featuring different depth sensitivities: 1–2 nm for AEY, 2–5 nm for TEY and 50 nm for FY [50]. In most cases, these signals can be detected concurrently for a depth-dependent electronic structural analysis. To study the electronic structures of nickel in NMC materials, Lin *et al.* performed Ni *L*-edge XAS at different states of charge (SOC) of the first cycle (Fig. 4b) [50]. The Ni *L_3_*-edge splits into high-energy (*L*_3-high_) and low-energy (*L*_3-low_) peaks as a result of Ni 2*p* – Ni 3*d* electrostatic interaction and the crystal field effects. The ratio between *L*_3-high_ and *L*_3-low_ is positively correlated with the nickel's oxidation state. By comparing the AEY, TEY and FY signals, a depth-dependent variation in nickel's oxidation state was observed in the charged electrode (Fig. 4b). The reduced Ni cation on the surface of the NMC cathode is often attributed to the surface reconstruction effect, which refers to the transformation of layered structure into spinel/rock salt structures. A follow-up study by the same group highlights that the liquid electrolyte plays a significant role in the surface reconstruction process and that the Ni is more prone to reduction at higher SOC [51]. The application of this depth-dependent sXAS approach was further extended to studying the thermal stability of LTMO, which is relevant to the lattice structure degradation, oxygen release and exothermic side reactions that could provoke a battery thermal runaway. In this respect, sXAS can reveal the thermal decomposition paths and the related degradation mechanisms. Alvarado *et al.* showed the evidence of reduction of Co and Ni in delithiated LiNi_0.8_Mn_0.1_Co_0.1_O_2_ (NMC811) after heating using Co and Ni sXAS spectra [52]. The intertwined and depth-dependent redox reactions of different TM cations highlight the complexity in the thermally induced cathode degradation, which is relevant to both the battery performance and safety.

Although there are a few nice demonstrations in the literature [53,54], the limited penetration capability of soft X-rays sets practical constraints on the experiment, in particular, for *insitu*/*operando* types of studies. In this regard, hard X-ray absorption spectroscopy demonstrates considerable advantages. The features in the transition metal *K*-edges’ spectra can provide information on the electronic and local lattice structures. This is widely used to probe the chemical valance state of the TM elements in LTMO. Depending on the scanned energy window, hard XAS includes XANES and EXAFS, the former is sensitive to the materials’ electronic structures and the latter is often used to probe the lattice structural information and was discussed in the previous section. Pioneered by Yang *et al.*, *insitu* XANES has been employed to study the evolution of TM elements in LTMO during the cycling process [55]. A comprehensive study of the Ni, Co and Mn in LiNi_1/3_Mn_1/3_Co_1/3_O_2_ (NMC333) using *insitu* XANES was reported by Yoon *et al.* [56], in which the *insitu* cells were charged up to 5.1 V at a charging rate of 0.05 C while the XANES data over the *K*-edges of Mn, Ni and Co were collected at set intervals (Fig. 4c). As the Li ion is deintercalated from the cathode, the Mn XANES spectrum changes its shape without apparent energy shift, while the Ni XANES spectrum shifts toward a higher energy, hinting that the average oxidation state of Ni increases and that of the Mn cation is relatively stable during the charging process. Interpretation of the Co *K*-edge XANES data is a bit more complicated because it is not obvious whether the subtle differences in the spectra are caused by the energy shift or by the line-shape deformation [57]. From a more fundamental perspective, the features in the XANES spectra are ultimately associated with transitions between different electronic states [56]. In the normalized *K-*edge XANES spectra, the weak pre-edge absorption is associated with the electric dipole-forbidden transition of a 1*s* electron to an unoccupied 3*d* orbital. The first shoulder features (to the left of the main absorption edge) are assigned to a shakedown process, which involves the 1*s* → 4*p* transition and is followed by ligand-to-metal charge transfer. The features in the main absorption edge are attributed to the purely dipole-allowed 1*s* → 4*p* transition. A more advanced approach for quantifying the XANES data is to conduct a comprehensive and thorough fitting of the entire XANES energy region in terms of a well-defined set of structural parameters using a method called MXAN [58]. However, this method requires a fairly well-defined model and is relatively more time-consuming and computationally expensive. Generally, XANES is a powerful method for analyzing the valence state of the TMs in the bulk of the LTMO cathode. The interpretation of XANES data is usually straightforward but might require extra effort when the changes in the spectrum features are subtle.

In all the above discussed spectroscopy techniques, the sample's response to the incident X-rays at a given energy is measured as a scalar. By adding energy resolving power to the detector for an XAS measurement, an additional dimension of the data is extended and more detailed chemical information can be retrieved. Resonant X-ray emission spectroscopy (RXES), often called RIXS, can probe electronic, orbital, magnetic and lattice excitations for the targeted element [53]. The photon-in-photon-out (PIPO) test mode makes RIXS bulk-sensitive and its probing depth can reach hundreds of nanometers in soft X-ray regime. The detector's energy resolving power makes it possible to reject unwanted photons and to single out the signal from a specific chemical state. More specifically, the total fluorescence yield (TFY) mode of sXAS may suffer from a signal mixing effect. For example, the TFY sXAS over the Mn *L*-edge contains a fairly strong contribution from oxygen, which leads to a distortion of the spectral line-shape. Unlike TFY sXAS, the partial fluorescence yield (PFY) and inverse partial fluorescence yield (iPFY) extracted from the RIXS map, can probe the bulk Mn chemical states without any line-shape distortion. To further demonstrate the rich information in a RIXS map, we show in Fig. S3 the Mn *L*-edge RIXS data acquired using a superconducting transition edge sensor (TES) detector at beamline 10–1 of SSRL. With energy resolution for both the incoming X-rays and the detected X-rays, the RIXS maps can be used to extract spectroscopic signatures in different modalities, for example TFY, PFY, iPFY and XRF, opening up vast opportunities. It has also been demonstrated that RIXS can be sensitive to anion redox reaction (ARR), which often overlaps with the transition metal signals and, therefore, could be challenging to investigate using the conventional sXAS approach. ARR, for example oxygen redox, widely exists in both Li-rich and conventional LTMO. Oxygen redox could offer extra capacity upon deep delithiation of the cathode. Its reversibility during the electrochemical cycling is often far from satisfactory, however. Despite tremendous efforts in this field, there is still a lack of conclusive understanding of the fundamental mechanism for the oxygen redox reactions. The core question of the oxygen redox mechanism rests on O^2–^ is oxidized in which form. In 2016, Luo *et al.* proposed a charge compensation mechanism for oxygen redox in a Li_1.2_Ni_0.13_Co_0.13_Mn_0.54_O_2_ cathode [59]. Upon deep delithiation, O atoms coordinated with Mn^4+^ and Li^+^ could generate localized electron holes near the top of the O^2–^ valence band, which compensated the charge and suppressed O release from the lattice. House *et al.* suggested formation of molecular O_2_ during the oxygen redox in Li-rich materials [60,61]. Upon charging, O^2–^ was oxidized to molecular O_2_. When occurring on the particle surface, this process is accompanied by layered-to-disordered phase transition. In the bulk, however, the formed O_2_ is trapped in vacancy clusters and can be reduced back to O^2–^ upon discharging. The order-disorder rearrangement is irreversible, causing a change in the coordination environment around O and leading to the voltage hysteresis. In addition, other mechanisms such as reductive-coupling-assisted formation of peroxides (O_2_^2−^) [62], formation of O–O peroxo-like dimers [63] and lattice O^n–^ (n < 2) [48] have also been proposed to explain the oxygen redox behavior in the Li-rich materials. An in-depth discussion on oxygen redox is beyond the scope of this review. More comprehensive pedagogical descriptions of the oxygen redox can be found in recent literatures [64–66]. Here we focus on discussing how RIXS reveals the oxygen redox in the LTMO materials. As is shown in Fig. 4d, soft X-ray RIXS over the oxygen *K-*edge was used to probe the evolution of oxygen redox activity in Li_1.17_Ni_0.21_Co_0.08_Mn_0.54_O_2_, a model Li-rich layered cathode [67]. In the pristine and 4.35 V-charged electrodes, excitation into the unoccupied TM 3*d* – O 2p^*^ (*E*_excitation_, 528–533 eV) and TM 4*sp* – O 2p^*^ (*E*_excitation_, > 533 eV) states results in similar emission energy profiles (*E*_emission_, 522–527 eV), corresponding to a decay from the delocalized oxygen valance band states to fill the excited O 1*s* core hole. However, for the 4.6 V-charged electrode, a new emission feature at 523.25 eV emerges. It is clearly distinguished from the TM-O hybridized states and can be attributed to the excitation to the new unoccupied O redox state at 530.8 eV. This feature is considered to be direct evidence of the lattice oxygen redox. Interestingly, this O redox feature reversibly appears even after 500 cycles, suggesting that the anion redox in 3*d* Li-rich NMC could be active over long-term cycling. Recently, Li *et al.* investigated the oxygen redox behaviors in Li-rich NMC under a mildly elevated temperature (up to ∼100°C) [68]. A bulk-oxygen-to-surface-TM charge transfer was observed at ∼100°C. This observation indicates thermally driven redistribution of lithium-ions. In addition to the Li-rich NMC cathode, conventional LiNi_1/3_Co_1/3_Mn_1/3_O_2_ and LiNiO_2_ are also reported to show the anion redox feature at high potentials [69,70]. Understanding the anionic redox mechanism can critically inform the designs for next-generation high voltage LTMO cathodes and the RIXS technique has demonstrated unique capability in unraveling the anionic activity with superior sensitivity and accuracy. It is worth pointing out that theoretical modeling of the RIXS data could greatly assist the interpretation. Compared with the existing modeling effort for conventional XAS, there are a lot of opportunities for developing novel modeling tools in RIXS.

## SYNCHROTRON CHARACTERIZATION OF LTMO’S MORPHOLOGICAL COMPLEXITY AND EVOLUTION

We elaborated on the multi-scale and high-dimensional morphological complexity in LTMO-based lithium-ion batteries at the beginning of this article (see Fig. 1d). Here we reiterate that such a hierarchical structure entails complicated function and degradation mechanisms that collectively govern the behaviors of the LIB across a broad range of time and length scales. This has motivated many synchrotron-based imaging studies on batteries from different perspectives. By virtue of high brightness and low beam emittance, synchrotron X-ray has demonstrated prominent advantages in non-destructive and high-dimensional imaging, which endow the capability of characterizing LTMO-based LIB with different spatial and temporal resolutions and with structural, compositional and chemical sensitivities. In the Supplementary data, section IV, we discuss a few examples of synchrotron-based imaging study of LIB from the cell scale down to the particle level. These studies target different phenomena that are of both scientific and industrial significance to battery research and serve as good examples to highlight the effectiveness of synchrotron-based imaging techniques in battery research.

We have already touched on redox heterogeneity in the LTMO battery cathode in the previous section, here we dive deeper into this topic and will highlight the interplay between the redox and lattice heterogeneities in the later part of this section. While the redox heterogeneity is ubiquitous in LTMO materials and is often regarded as an unwanted phenomenon because it tends to escalate asymmetric stress and cause structure disintegration [71], we highlight that engineering the particle with patterned redox heterogeneity can potentially improve the cycling stability. For example, incorporation of zirconium into the cathode as a coating layer or a trace dopant could effectively modulate the surface heterogeneity for a single-crystal NMC particle and suppress the interfacial lattice reconstruction, benefiting the electrochemical stability at high cut-off voltage [72].

**Figure 5.**
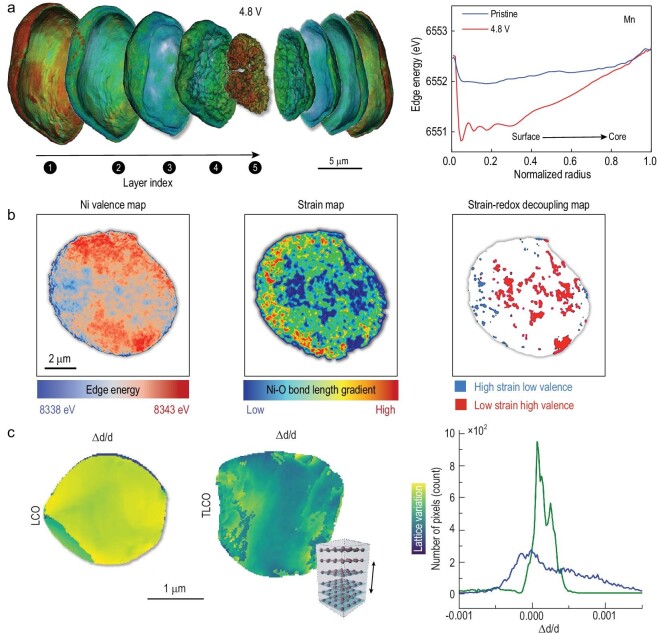
Synchrotron-based X-ray imaging studies of redox and lattice heterogeneities in LTMO cathode materials. (a) The depth-dependent redox stratification effect in a Li-rich NMC (Li_1.2_Ni_0.13_Mn_0.54_Co_0.13_O_2_) cathode. Adapted with permission from [73], Copyright 2020, Springer Nature. (b) The inhomogeneous redox and lattice strain in cycled LiNi_0.8_Mn_0.1_Co_0.1_O_2_ particle. Adapted with permission from [76], Copyright 2021, American Chemical Society. (c) Titanium doping effect on lattice variation in the LiCoO_2_ particle. Adapted with permission from [77], Copyright 2020, Elsevier.

We choose to highlight two studies that elucidate evolution of the redox heterogeneity in LTMO as a function of the electrochemical states. The first example is a recent study by Zhang *et al.* on the depth-dependent redox stratification effect in a Li-rich NMC (Li_1.2_Ni_0.13_Mn_0.54_Co_0.13_O_2_) cathode [73]. The complexity in this material lies with the co-existing and intertwined cation and anion redox reactions upon charging to high voltage. Involvement of the lattice oxygen anions in the redox reaction has been reported using advanced synchrotron spectroscopic tools, for example RIXS, which reveals the electronic state of the oxygen anion when the cathode is deeply delithiated, as we elaborated in the previous section of this article, without offering spatial resolution to probe the chemical heterogeneity. The authors employed the nano-resolution spectro-tomography method to probe evolution of the depth-profiles for the oxidation states of all three transition metal cations in the system (Mn, Co and Ni). While an anticipated depth-dependent particle polarization effect was observed in the particle that was charged to a low voltage of 4.3 V, surprising nonmonotonic depth profiles were found in material that was charged to 4.8 V (Fig. 5a), with the core and the outermost layer of the fully charged particle showing high Mn valence and the Mn in the transition layers being relatively reduced. Further testing revealed that the valence of Ni gradually increases from the particle surface to the core region of the fully charged particle and that Co's valence state has a similar distribution tendency to that of Mn's valence state. It has been reported that the oxygen redox reaction in a Li-rich LTMO cathode onsets at around 4.4 V. Therefore, development of the nonmonotonic redox profiles for the transition metal cations was attributed to the involvement of oxygen activity, which also demonstrates a depth-dependent profile that can be inferred indirectly from the behaviors of the transition metal cations. While this example highlights the difference in the spatial profile of the chemical heterogeneity at different electrochemical states for the LTMO cathode, an experimental observation of the LTMO particles under *insitu* conditions would be viewed as a desirable improvement to this study, in particular, for capturing the dynamic evolution over repeated electrochemical cycling. This has motivated a range of synchrotron-based *insitu* and *operando* imaging studies, including the second example that we will cover next. In work by Xu *et al.*, a single LiCoO_2_ particle inside a working pouch cell was monitored as the cell underwent a designed electrochemical cycling process [74]. An unambiguous inverse correlation between cycling C rate and the recovery rate was reported for this particular particle, highlighting that a single cathode particle is able to respond differently to different externally applied reaction driving forces. In addition to the rate dependence, the rearrangement of the SOC spatial pattern also changed under stabilized and repeated cycling conditions (0.2C, from the 3rd to the 21st cycles). The results indicated that, in addition to the electrochemical redox heterogeneity being highly dependent on the C rate, an electrochemical reactivation process with a small C rate can potentially be designed to recover some of the lost capacity from previous fast charging operations. This type of *insitu* and *operando* synchrotron spectro-imaging measurement has been employed to study the correlation between redox and morphology (size and crystal plane [75]) of the cathode particles under high C rate. These insights are valuable to the design of high-power-type LTMO materials.

While the redox heterogeneity is pervasive in LTMO materials, it is often attributed to the morphological complexities, the inhomogeneous distribution of Li ions and, thus, the local SOC variations. Here we point out that the atomic structural arrangements, including the lattice distortions and defects, have a very significant role in affecting the redox heterogeneity and the battery performance. These lattice heterogeneities could be induced by imperfect nucleation and crystallization processes during the synthesis, they could also be induced by undesired side reactions that occur during the battery operation. Synchrotron offers various experimental probes with sensitivity to the material's lattice arrangements, for example XRD, EXAFS and PDF, which we have covered in the previous designated section. Here we discuss experimental approaches to resolve the lattice heterogeneity in LTMO at the mesoscale with nano-resolution. In a recent development that combines the full-field nano-resolution transmission X-ray microscopy/tomography with the EXAFS scan, Qian *et al.* visualized and correlated the redox features and the lattice bonding features in a cycled poly-crystalline NMC particle (Fig. 5b) [76]. In the presented TXM-EXAFS measurements, the authors extracted the Ni's valence state from the XANES region and the Ni−O bond length from the EXAFS region. To address the challenge of low signal-to-noise in the EXAFS spectra, the authors formulated a machine-learning-assisted data clustering approach for reduction of the noise and the dimensionality of the data. Upon recovering a map of the Ni−O bond length over the particle, the authors further quantified the amplitude of the gradient, which was then attributed to a strain-induced lattice distortion. While a general position correlation between the redox and the strain was observed, in good agreement with the common wisdom, localized regions with a strain-redox decoupling effect were also observed and attributed to different degradation mechanisms. Combined with 3D nano-tomography, it was found that the regions of high Ni valence and low strain are likely detached from the particle because of cracks, and no longer participate in the electrochemical reaction. The regions of low Ni valence and high strain, on the other hand, populate on the particle surface and may be the result of surficial lattice reconstruction. This redox-strain coupling/decoupling behavior features the complexity of degradation mechanisms in LiNi_0.8_Co_0.1_Mn_0.1_O_2_ materials upon prolonged battery operation. This study largely benefits from the correlation analysis of the redox and lattice heterogeneities in the LTMO cathode.

Another approach to directly reveal the lattice heterogeneity is to conduct a spatially resolved XRD measurement using a small X-ray focal spot. For this purpose, the hard X-ray nanoprobe (HXN) has demonstrated exceptional capability of detecting the lattice structures and their variations in space [77]. In an HXN study of quasi-single-crystalline LTMO material, the X-rays were focused to a spot of ∼30 nm onto a sample, producing a spotty diffraction pattern with peaks in the orientations that satisfy the Bragg conditions. The sample can be rocked over a small angular range and a 2D raster scan is often conducted to collect the signals from different parts of the sample. In a recent work by Hong *et al.*, the authors investigated the Ti dopant's impact on the structure of LiCoO_2_. They conducted HXN-based mapping to detect the degrees of lattice distortions in Ti-dopped LiCoO_2_ and in bare LiCoO_2_ [77]. Their results (Fig. 5c) suggest that incorporation of a trace amount of Ti into the LiCoO_2_ lattice has significantly distorted the host lattice because of the incompatibility of the dopant with the LiCoO_2_ matrix. The heterogeneous lattice configuration increases the energy barrier of the anisotropic micro-strain in the LiCoO_2_ particle during delithiation to 4.6 V, suppressing undesired O3 to H1-3 phase transition, as evidenced by the improved stability of the Ti-doped LiCoO_2_ cathode.

Coherent diffractive X-ray imaging is another method that has demonstrated some success in evaluating the evolution of lattice defects in LTMO upon battery operation. Singer *et al.* studied the nucleation of dislocation defects and their dynamics in lithium-rich LTMO cathode materials during battery charging using a Bragg coherent diffractive imaging (BCDI) technique [78]. In their experiment, a rather sophisticated phase retrieval method was used to reconstruct the dislocation networks inside the particle from the Bragg diffraction pattern. As the cathode was charged to a higher voltage, more dislocation defects were formed, which could be associated with the oxygen evolution. Unfortunately, this effect prevented the authors from evaluating the particle at a voltage above 4.4 V because the phase retrieval method failed at the state with abundant lattice defects. Although this is an exciting development with promising future, the technical challenges associated with coherent X-ray imaging must be addressed for practical and routine applications of this method.

## CORRELATIVE SYNCHROTRON TECHNIQUES AND SCIENTIFIC DATA MINING

In LTMO-based battery material, the electrochemical delithiation/lithiation process is rather intricate, involving morphology and composition effects, electronic and lattice structural evolutions and sophisticated interplay between the active material and inactive components, for example carbon, binder and electrolyte. An in-depth understanding of this process requires thorough and systematic investigation into different aspects of the material. In the previous sections, we elaborated on the respective strength of various synchrotron-based experimental techniques. Here we highlight that performing these characterizations in a correlative manner can provide unique insights that are otherwise inaccessible. This is particularly the case for the multi-modal imaging of battery materials and we choose to briefly discuss two examples of this kind below.

**Figure 6.**
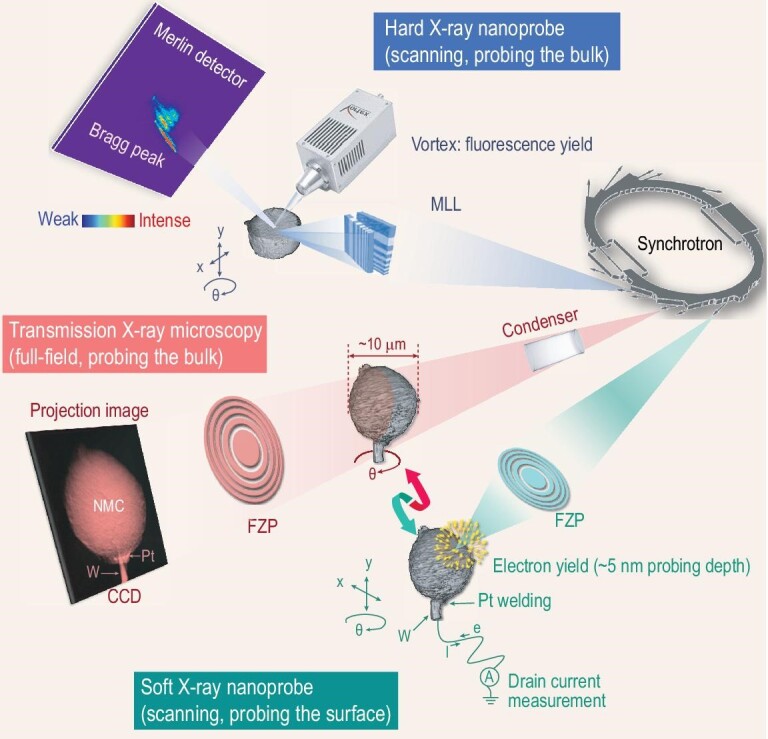
Synchrotron-based correlative analysis of LTMO cathode materials. Adapted with permission from [79], Copyright 2020, Springer Nature. Adapted with permission from [72], Copyright 2021, Elsevier.

While it is broadly appreciated that the battery charging/discharging is ultimately a bulk reaction but that the surface chemistry has an important role to play in this process, a detailed picture and a fundamental understanding of this surface-to-bulk interaction is rather elusive because of a lack of direct experimental observations. In work by Li *et al.*, a single poly-crystalline LiNi_0.8_Mn_0.1_Co_0.1_O_2_ particle was imaged correlatively using both soft and hard X-ray probes with nanoscale spatial resolution (see illustration in Fig. 6) [79]. The hard X-ray nano-tomography reconstructs the 3D internal structure of the particle non-destructively. Meanwhile, the soft X-ray nanoprobe can precisely map out the surface chemical distribution, elucidating the heterogeneous surface reconstruction and passivation effect. Correlating these two measurements, it was observed that the regions with higher porosity in the bulk tend to suffer more severe surface lattice reconstructions. This experimental observation informed systematic finite element modeling that revealed the coevolution of Li distribution, stress and the morphological damage in the NMC secondary particle.

Qian *et al.* combined the nano-resolution full-field hard X-ray spectro-tomography and the scanning hard X-ray nanoprobe techniques to investigate a single-crystalline NMC particle with a trace amount of zirconium doping [72]. By correlating the dopant and the SOC distributions at the mesoscale, the authors revealed an interesting pattern of Ni redox inhomogeneity, which suggests that the mesoscale electrochemical kinetic is modulated by facet-selective Zr segregation. It is useful to highlight that this study leverages the high sensitivity of the scanning nanoprobe and the high efficiency of the full-field spectro-tomography. By putting these two datasets together, it is revealed that the lithium diffusion path parallel to the (003) plane was blocked by a Zr-rich layer segregated on the side edge of the particle, forming kinetically favored particle corners. This result is helpful to understand the dopant's influence and to design novel LTMO materials with engineered compositional arrangements. Considering the limited resolution of synchrotron-based imaging analysis, combination with electron microscopy could be an effective way to obtain comprehensive chemical and structural information of samples. X-ray microscopy can provide chemical, electronic and magnetic information of the material, while electron microscopy could quantify the elemental composition with atomic-resolution [80]. A correlative combination of these complementary techniques could offer valuable multi-modal experimental data that are critical to understanding of the functioning and degradation mechanisms of the battery materials.

Finally, we would like to bring the reader’s attention to a new research direction that combines state-of-the-art synchrotron experimental techniques with novel computational developments, for example machine learning and data mining, for battery studies. In general, these novel computational methods can greatly assist battery researchers and synchrotron scientists in reducing large-scale experimental data and extracting scientifically important information. Machine learning approaches have been used to guide high-throughput synchrotron experiments for material screening [81], to conduct classification and dimension reduction for chemical imaging data for capturing undesired side reactions in batteries [76,82,83], to assist with battery imaging data processing, segmentation and quantification [73,84] and to construct a numerical model for battery failure and lifetime prediction [85,86].

**Figure 7.**
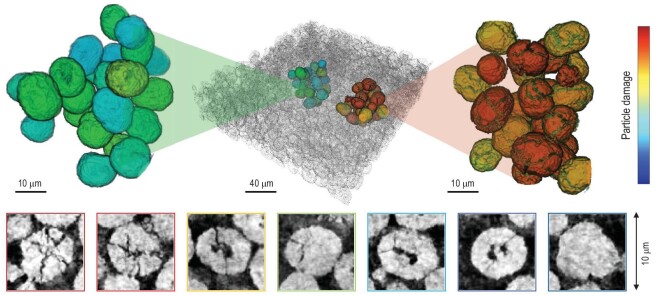
Quantification for NMC particle cracking in real-world composite cathode. These particles exhibit very different cracking patterns (bottom row). The particle clusters also demonstrate different degrees of structural disintegration (top row).

For example, to evaluate the chemomechanical breakdown of LTMO cathode particles, great care is required in the statistical analysis to avoid any pitfalls in interpretation of the imaging data. This is because the damage pattern is highly heterogeneous and particles within the same electrode can exhibit very significant differences in their respective degrees of cracking. As an example, we show in Fig. 7 the synchrotron nano-tomographic results of a NMC composite cathode that is recovered from a fast-cycled coin cell. Within this electrode, the particles exhibit very different cracking patterns as demonstrated by the selected slice-views shown in the bottom row of Fig. 7. The image frames in the bottom row of Fig. 7 are color-coded to the degree of damage in the corresponding particles, which is determined by quantifying the crack-surface areas within these particles. Moreover, the particle clusters can also demonstrate different degrees of damage, indicating differences in their engagement of the electro-chemo-mechanical processes. The two selected clusters, only ∼50 μm apart, are very different in structural integrity. It is worth pointing out that, for this dataset, the traditional image segmentation approach has low accuracy in identifying and segmenting the severely cracked particles. Machine learning methods can offer much needed assistance in identifying and quantifying the thousands-to-millions of particles in the electrode, with superior accuracy and efficiency. Evaluation of the massive number of NMC particles (shown in Fig. 7) largely benefits from such a development by Jiang *et al.*, with the source code made openly available to the research community [87].

This is a highly interdisciplinary and rapidly evolving research field. We anticipate significant growth in research endeavors in this direction from researchers of different backgrounds.

## CONCLUSION AND PROSPECTS

Rechargeable battery technologies have demonstrated a tremendous impact on modern society, facilitating groundbreaking developments in consumer electronics, electric vehicles and grid energy storage, all of which have enormous market value. A practical battery is a highly complicated multi-component device, featuring structural and chemical evolutions at different time and length scales upon battery operation. These battery components work in a synergistic way to fulfill the energy storage functionality. There are very active research efforts looking into different battery components, but, at present, the cathode material remains the major bottleneck for improving energy density. Therefore, we chose LTMO, a group of leading cathode materials, as our focus for this review and looked into the complicated thermo-electro-chemo-mechanical interplay in the LTMO cathode.

When studying such a material system with enormous structural and chemical complexities, advanced characterization tools are indispensable. In particular, the study of a practical LTMO cathode with intertwined microstructural, compositional and chemical heterogeneities needs a holistic suite of experimental methods with different sensitivities. Synchrotron techniques stand out as a suite of effective methods for non-destructive battery characterization with sensitivities to the lattice, electronic and morphological structures. In this article, we discuss the complexity and evolution of the LTMO cathode's structural and chemical properties under different working conditions. We further review relevant synchrotron-based research works that address the frontier challenges in this field and provide novel insights for understanding the battery degradation mechanisms and for informing the effort to improve the battery cathode.

This is a highly active research field. State-of-the-art developments involve efforts from material science, synchrotron development and advanced computing. In our review, we included a few recent works that integrate these cutting-edge developments, for example *insitu*/*operando* battery studies at synchrotron, cross-beamline and cross-facility multi-modal synchrotron studies for battery cathode, machine learning and data mining assisted battery studies at synchrotron. These case studies serve as good examples to highlight the interdisciplinary nature of this research field. While the synchrotron characterization techniques could offer valuable insights into the battery research, there are some drawbacks that have to be considered. For example, the radiation damage could potentially jeopardize the study, especially the long-time *insitu/operando* experiments. X-ray induced damage can come in different forms and, therefore, a careful dose management plan is often needed for the experiment. In addition, the spatial resolution of X-ray microscopy is often limited to a-few-nanometers to tens-of-nanometers level, which is insufficient to visualize some of the localized features. Therefore, a multi-modal approach incorporating other high resolution characterization tools, for example scanning transmission electron microscopy with atomic resolution [88], would be beneficial. Another example is the correlation of neutron tomography with X-ray tomography [89]. The interaction of neutrons with the materials could offer unique sensitivity to elements (e.g. lithium) that are hard to probe using X-rays.

Looking forward, we envision that the future research challenges in this field will be associated largely with the growing complexity in novel battery materials and systems. In particular, the battery materials’ dynamic transformation, involving metastable phases that are functionally critical, is often overlooked in conventional studies, and could be important for novel battery material design. Such a complexity can be further amplified as the batteries are operated under extreme conditions for different applications, for example ultra-fast charging, cold/hot climate and space exploration. The ongoing and projected major upgrades of the synchrotron and free electron laser (FEL) facilities bring scientific opportunities to this research field. Enhancements in X-ray brightness and brilliance could facilitate X-ray experiments with unprecedented resolution and sensitivity, critical to investigation of next-generation battery materials. As a concluding remark, we are confident that, with collaborative efforts from material scientists, synchrotron scientists and computational scientists, this community is making substantial impacts on the energy storage technology and moving toward a global energy solution with much desired sustainability.

## Supplementary Material

nwab146_Supplemental_File
