# Supplementary material for: Structural and chemical evolution in layered oxide cathodes of lithium-ion batteries revealed by synchrotron techniques
Source: Natl Sci Rev. 2021 Aug 17;9(2):nwab146. doi: 10.1093/nsr/nwab146 (PMC8824737; doi:10.1093/nsr/nwab146)
Supplement: nwab146_Supplemental_File [file nwab146_supplemental_file.docx]

Supplementary Information for

Structural and chemical evolution in layered oxide cathodes of lithium-ion batteries revealed by synchrotron techniques

Guannan Qian (钱冠男)^1,2, †^, Junyang Wang (汪君洋)^1,3, †^, Hong Li (李泓)^3^, Zi-Feng Ma (马紫峰)^2^, Piero Pianetta^1^, Linsen Li (李林森)^2,4,*^, Xiqian Yu (禹习谦)^3,*^ and Yijin Liu (刘宜晋)^1,*^

^1^ Stanford Synchrotron Radiation Lightsource, SLAC National Accelerator Laboratory, Menlo Park, CA 94025, USA

^2^ Department of Chemical Engineering, Shanghai Electrochemical Energy Device Research Center (SEED), School of Chemistry and Chemical Engineering, Frontiers Science Center for Transformative Molecules, Shanghai Jiao Tong University, Shanghai 200240, China

^3^ Beijing Advanced Innovation Center for Materials Genome Engineering, Key Laboratory for Renewable Energy, Beijing Key Laboratory for New Energy Materials and Devices, Institute of Physics, Chinese Academy of Sciences, Beijing 100190, China

^4^ Shanghai Jiao Tong University Sichuan Research Institute, Chengdu 610213, China

^5^ These authors contributed equally: Guannan Qian, Junyang Wang

^*^[linsenli@sjtu.edu.cn](mailto:linsenli@sjtu.edu.cn); [xyu@iphy.ac.cn](mailto:xyu@iphy.ac.cn); [liuyijin@slac.stanford.edu](mailto:liuyijin@slac.stanford.edu)

**Abbreviations**

AES: Auger electron spectroscopy

ARR: anion redox reaction

BCDI: Bragg coherent diffractive imaging

CBD: carbon-binder domain

EXAFS: extended X-ray absorption fine structure

EV: electric vehicle

FEL: free electron lasers

FT: Fourier transformed

FY: fluorescence yield

HAXPES: hard X-ray photoelectron spectroscopy

HXN: hard X-ray nanoprobe

iPFY: inverse partial fluorescence yield

LIB: lithium-ion battery

LTMO: layered transition metal oxides

NMC: lithium nickel manganese cobalt oxides

PDF: pair distribution function

PFY: partial fluorescence yield

RIXS: resonant inelastic X-ray scattering

RMC: reverse Monte-Carlo

SOC: state of charge

SSRL: Stanford Synchrotron Radiation Lightsource

STEM: scanning transmission electron microscopy

sXAS: soft X-ray absorption spectroscopy

TES: transition edge sensor

TEY: total electron yield

TFY: total fluorescence yield

TR-XRD: time-resolved X-ray diffraction

TXM: transmission X-ray microscopy

XAFS: X‐ray absorption fine structure spectra

XANES: X-ray absorption near edge structure

XAS: X-ray absorption spectroscopy

XES: X-ray emission spectroscopy

XPS: X-ray photoemission spectroscopy

XRD: X-ray diffraction

XRF: X-ray fluorescence

- 1. **Brief introduction of synchrotron characterization techniques.**

When X-rays are delivered to the sample, they interact with the matter in a few different ways at different likelihood, leading to several signals that are associated with different material properties, respectively. Generally speaking, absorption, phase-shifting, and scattering are three different basic interactions between the X-rays and the matter, which derive various X-ray characterization techniques that are sensitive to the lattice structure, electronics structure, and the micro-morphology. X-ray microscopy, spectroscopy and diffraction/scattering are often regarded as three major experimental modalities of synchrotron techniques. They, however, can be combined, forming more advanced methods, e.g., spectro-microscopy, diffractive imaging, and resonant scattering (see Fig. S1).


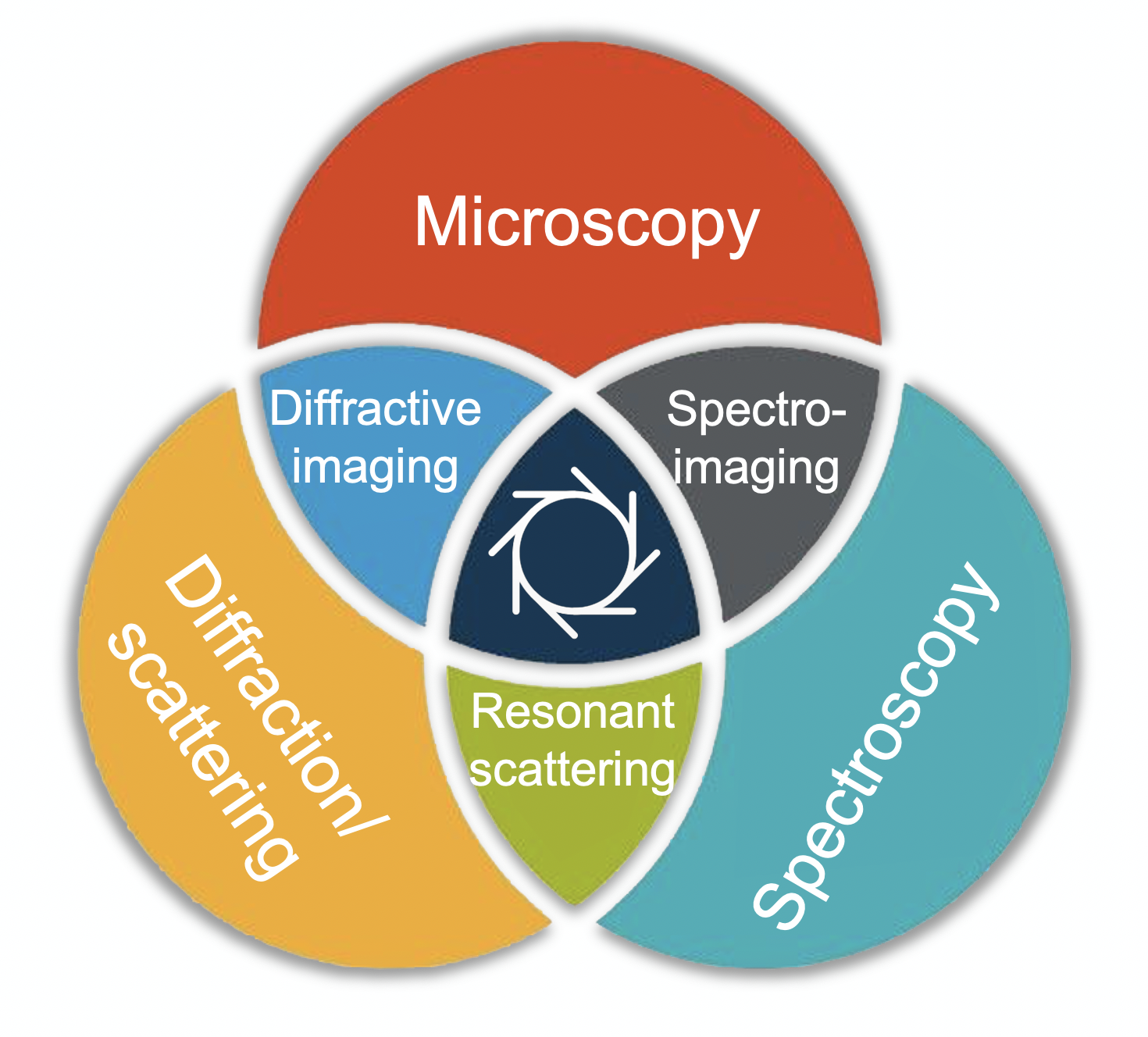


**Figure S1.** Illustrations of synchrotron experimental modalities and their combinations.

Absorption refers to the sample’s ability to attenuate the incident beam, which is modulated by the X-ray energy level as well as the matter’s density and electronic structures, formulating a suite of powerful X-ray absorption spectroscopic (XAS) techniques [1-3]. When an atom absorbs a photon, an electron, e.g., a photoelectron, is kicked out of its orbit, leaving the atom at an exited state. This process can be utilized to conduct X-ray photoemission spectroscopy (XPS), which is sensitive to the elemental composition, the chemical state, the electronic structure, and density of the electronic states over the sample surface [4-6]. The atom’s excited state is often unstable and the system will spontaneously reduce its energy by emitting fluorescence photons or Auger electrons, which facilitate the X-ray fluorescence (XRF) analysis or Auger electron spectroscopy (AES) [3, 7]. Scattering is a phenomenon, in which the incident photons are diffused by the matter, changing their direction and propagation. When there is no energy exchange between the photons and the matter, the scattering is elastic and fingerprints the material’s lattice structural properties [8, 9]. In the case of inelastic scattering (Compton scattering) [10, 11], the amount of exchanged energy often resonant with and excites the matter. By tuning the incident energy to a targeted absorption edge, it maximizes the cross section for inelastic scattering, opening up the opportunity for detailed electronic structural investigation using resonant inelastic X-ray scattering (RIXS) technique [12-14]. These synchrotron experimental techniques have different penetration depths. Collectively, they can probe the lattice structure, electronic structure, chemical valance state, and multi-scale morphology with high efficiency and precision, which are very relevant to the study of battery materials. More specifically, the change in the battery material’s crystalline structure can be monitored using X-ray diffraction (XRD), pair distribution function (PDF), and extended X-ray absorption fine structure (EXAFS) [15]. The states of the chemical and electrochemical redox reactions can be probe by evaluating the elements’ chemical valance states with X-ray absorption spectroscopy (XAS, including soft X-ray absorption spectroscopy (sXAS) and X-ray absorption near edge structure (XANES)), X-ray emission spectroscopy (XES), X-ray photoelectron spectroscopy (XPS), etc. [16] Advanced synchrotron microscopy techniques can further add 2D or 3D spatial resolution in different experimental configurations, including transmission X-ray microscopy (TXM), scanning micro-/nano-probes, ptychography, and tomography [17]. More recently, several of these techniques are integrated to conduct correlative multi-modal characterization that formulates a more holistic view of the structural and chemical complexity in the LTMO battery cathode under *ex-situ*/*in-situ*/*operando* conditions [18-21]. Another key development in this research field is the implementation of the advanced computational approaches. Impressive achievements and immense potential have been demonstrated using machine learning and data mining algorithms [22-24]. The novel data-driven developments critically complement the synchrotron experimental capabilities by offering the capability to extract the scientifically important information from the large-scale experimental data effectively and efficiently with greatly reduced human labor and error. We briefly summarize the characteristics of these X-ray techniques used for battery research in Table S1.

**Table S1**. The synchrotron-based X-ray techniques for LTMO investigations.

| **Technique** | **Detection modality** | **Contrast mechanism** | **Temporal resolution** |
| --- | --- | --- | --- |
| XRD | Reflection mode  Transmission mode | Lattice deformation and phase transformation | Seconds |
| PDF | Total scattering | Short-range ordering | Seconds to hours depending on the Q range and step size |
| EXAFS | Transmission mode  Fluorescence mode | Local coordination environment | Seconds to minutes |
| sXAS | Transmission mode  Fluorescence mode  Electron mode | Chemical and electronic structure (Charge, spin, orbital) contrast | Seconds to minutes |
| XANES |  |  |  |
| RIXS (mapping) | Scattering/inelastically scattered photon energy | Charge, spin, orbital, and lattice excitations | Minutes to hours depending on TM or O elements |
| XPS | Electrons | Binding energy contrast | Seconds to minutes |
| Micro/nano CT | Full-field imaging | Micro/nano morphology based on absorption/phase contrast | Seconds (micro-CT) to minutes (nano-CT) |
| Micro/nano probe | Scanning imaging | Compositional contrast, lattice deformation | Minutes to hours depending on the scan area and step size |

- 1. **Complexity and evolution in LTMO’s lattice structure.**

The traditional lithium-stoichiometric LiTMO_2_ (TM=Ni, Co, Mn, etc.) have a cubic close-packed oxygen framework with an AB-CA-BC stacking sequence (O3-type structure in the notation of Delmas [25]), exhibiting a rhombohedral α-NaFeO_2_ type structure with *R-3m* space group (Fig. S2a). In the structure, transition metal cations locate at octahedral 3b sites between oxygen layers, forming TM slabs consisting of edge‐sharing TMO_6_ octahedra, while lithium ions occupy the octahedral 3a sites in Li slabs, which can reversibly extract/insert from/to the Li sites between the TM slabs. Typical lithium-stoichiometric LTMO cathodes sharing the O3-type structure include LiCoO_2_, LiNiO_2_, and their derivatives, such as Li(Ni_x_Co_y_Mn_1-x-y_)O_2_ (NMC), and Li(Ni_x_Co_y_Al_1-x-y_)O_2_ (NCA). Lithium-rich LTMOs (Li_1+x_TM_1-x_O_2_), derived from the parent lamellar LiTMO_2_, are created by partially substituting transition metal cations with excess lithium ions on the TM slabs. As a result, it leads to the formation of a Li‐TM_6_ honeycomb‐type superstructure, which lowers the symmetry of material structure from 𝑅-*3*𝑚 to monoclinic *C2/m* symmetry. For example, Li_2_MnO_3_, alternatively noted as Li[Li_1/3_Mn_2/3_]O_2_, exhibits an O3-type layered structure with *C2/m* symmetry, which has one-third of Mn^4+^ in TM slab replaced by Li^+^ ions to form honeycomb Li-Mn_6_ superstructures (Fig. S2b). The superlattice ordering on the TM slabs results in the peculiar Bragg peaks in the range of ~ 20° to 35° (*Cu K_α_*). This in-plane ordering of Mn^4+^/Li^+^ cations also appears in other lithium-rich manganese-based LTMOs and forms local superlattice structure [26].


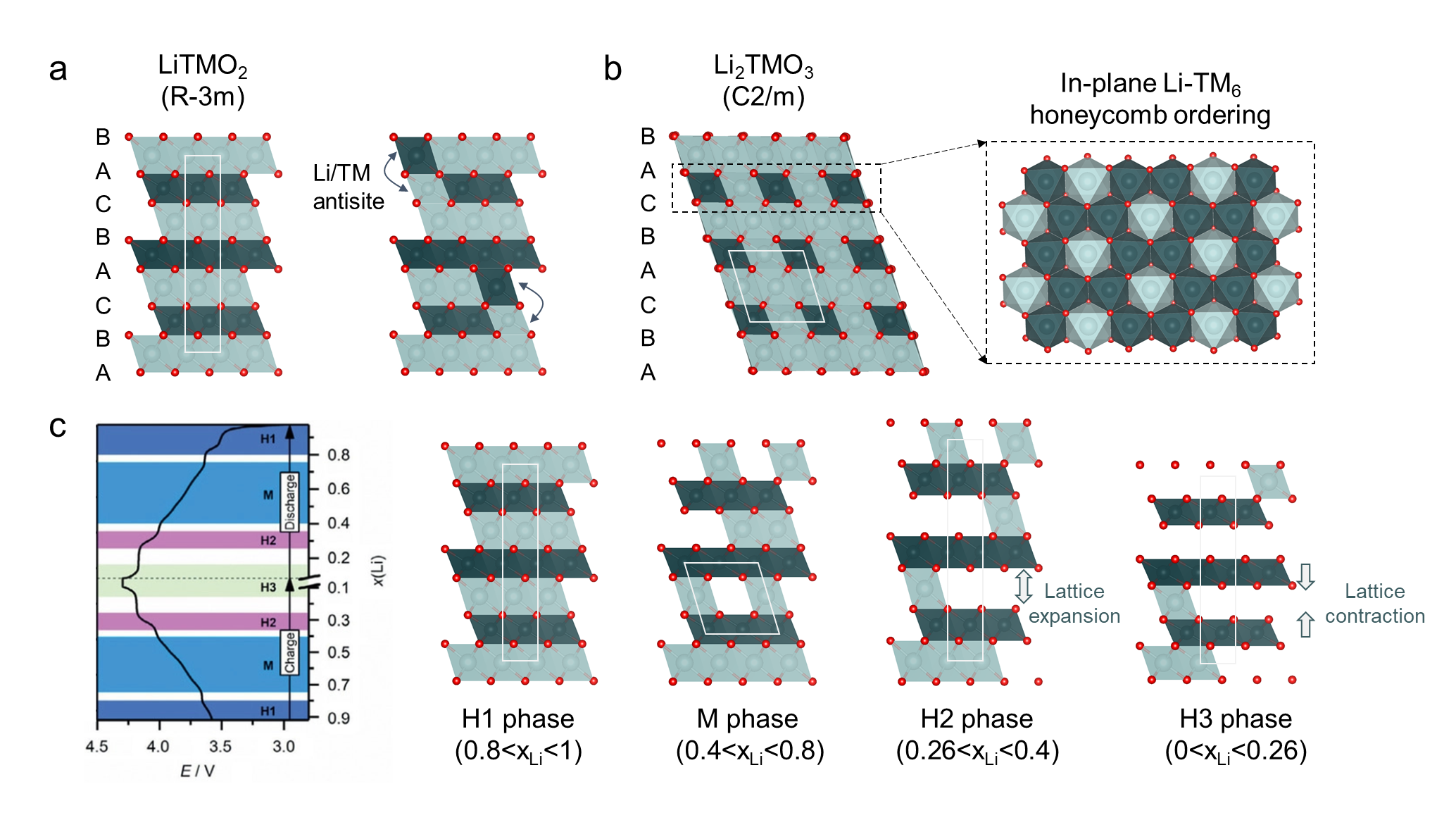


**Figure S2.** Illustrations of crystal structures relevant to the layered LTMO cathodes. (a) O3-type stacking sequence and cation antisite defects of LiTMO_2_ in the hexagonal lattice. (b) Monoclinic unit cell of lithium-rich LTMO cathode Li_2_TMO_3_ with an in-plane Li-TM_6_ honeycomb-ordering. (c) Lattice structure evolution of layered LiTMO_2_ cathodes during the first deintercalation process. Adapted with permission from [27], Copyright 2020, Wiley-VCH.

Other than the ideal scenario, where TM ions and Li-ions always occupy their specific sites in the LTMO’s lattice, crystalline defects also can be feasibly generated in the lattice structure, which significantly increase the structural complexity of LTMO. Noteworthily, those structural defects can play a key role in determining the electrochemical properties of LTMOs. For example, Li/Ni antisite defects, also referred to as “Li/Ni mixing” (or intermixing), are commonly observed in Ni-rich LTMOs such as LiNi_0.8_Mn_0.1_Co_0.1_O_2_ (NMC811). The Ni^2+^ ions sitting in the Li sites of Li slabs can affect the Li^+^ diffusion kinetics in the lattice structure and even cause structural collapse during the charging process as Ni^2+^ is oxidized to smaller sized Ni^3+^, which blocks the Li^+^ mobility. Though the Li/Ni antisite defects in LTMO are generally attributed to the similar size of Li^+^ (0.076 nm) and Ni^2+^ (0.069 nm) in most of the previous research works [28], it cannot explain the finding of increased amount of Li/Ni antisite defects in the Ni-based LTMOs with more Ni^3+^ (0.056 nm). Hence, Zheng *et al.* have proposed a new mechanism for the formation energy of Li–Ni antisite defects in terms of a super-exchange interaction between TM atoms [29]. It can be mainly affected by two factors: (1) the extent of structural distortion that can be quantified by the change of bond length after occupant’s exchange in the Li and TM sites and (2) the difference of super-exchange interaction between various configurations of TM^x+^-O^2-^-TM′^y+^, which changes with the formation of the antisite defects.

The antisite defects in the material structure can be generated not only during the material synthesis but also during the electrochemical cycling. For example, Shao-horn *et al.* reported the finding of cations migration from the transition-metal slabs to Li slabs during electrochemical cycles by using TEM analysis [30]. Independently, Kang *et al.* reported that the surface structure of LiNi_0.5_Co_0.2_Mn_0.3_O_2_ undergoes a phase transition from a O3 phase (*R-3m*) to a spinel-like structure (*Fd-3m*) or rocksalt phase (*Fm-3m*) because of the TM cations migration during electrochemical cycling [31]. At a deeply charged state, the layered structure of LTMO materials becomes extremely unstable due to the increased structural stress caused by the empty lithium sites. Thus, it prompts TM cation migration from TM layer to lithium layer. The TM migration tends to occupy every second Li vacancy to relieve the electrostatic repulsion between the disordered cations and antisite defects are thereby progressively accumulated in the material structure during an extended cycling process.

The TM_Li_-V_TM_ antisite defects induced by TM migration during charging have been identified experimentally using various analytical tools. It has been found that limited reversibility of intra-cycle TM migration results in the continuous growth of spinel-like disordered phases in the electrode materials, which is commonly associated with the voltage decay of the batteries [32]. Consequently, exacerbated voltage fade is found in Li-rich NMC upon extended cycling as more TM ions were trapped in the Li layer [33]. However, it is important to mention that the fundamental mechanism for the observed voltage decay is not essentially determined by the TM migration behaviors but the resulting confinement of TM ions in the Li layer [34], which suggest that a reversible TM migration behavior between TM layers and Li layers will not necessarily lead to the voltage decay of the batteries. Unfortunately, at a state of low Li stoichiometries for most lithium-rich LTMOs, TM migration to the Li layer is a thermodynamically favored and irreversible phenomenon during the charging process. As such, various approaches such as surface coating, cation doping, adding electrolyte additives, and composition tuning, have been used to mitigate the TM migration. A complete prevention of the voltage decay over long-term cycling, however, remains a daunting challenge.

Besides the antisite defects, lattice stacking disorder is another common structural defect in layered LTMOs, while the TM slabs might have an in-plane cation ordering. Thereby, it significantly increases the lattice structural complexity and the difficulties for structural characterization. First, the occurrence of stacking disorder defects causes diffuse scattering of the lattice structure and leads to a considerable broadening of the superlattice reflections. Second, different stacking sequences of the TM slabs along the *c* axis in LTMOs generate different lattice symmetry for the materials [35]. For example, Breger and Meng *et al.* have proposed two different ways of stacking in Li_2_MnO_3_, where A1-B1-C1 corresponding to *C2/m* and A1-B1-C2 corresponding to *P3_1_12* space groups [36]. Using these space groups, XRD patterns of Li_2_MnO_3_ can be well simulated and the *C2/m* stacking scheme is found to be better in evaluating the stacking fault probabilities.

Moreover, Meng *et al.* also pointed out that the generation of the *P3_1_12* lattice symmetry can be attributed to an abnormality of the *C2/m* sequence [37]. In parallel, Riou *et al.* have proposed an A1-B1-C2-A2-B3-C1 stacking sequence to explain the lattice structure of Li_2_MnO_3_ with a symmetry of the *C2/c* space group [38]. Such an inconsistency in their interpretation can be caused by the negligible energy difference between the proposed lattice sequence of the lattice structure, which is very sensitive to the synthesis temperature and thereby lead to random stacking faults in Li-rich LTMOs. Moreover, upon lithium removal from the lattice of LTMOs during charging, oxygen slabs tend to glide toward each other, and a structural rearrangement happens in the materials. As a consequence, a distorted ccp oxygen lattice (at different degrees) and a hexagonal close packing of oxygen can form in the lattice structure if a large portion of Li ions are removed. Therefore, a series of irreversible phase transitions are frequently observed in the LiTMO_2_ upon a deep extraction of Li-ion.

Specifically, pristine LiTMO_2_ first undergoes a solid‐solution reaction until the state of charge at ~Li_0.8_TMO_2_. It then proceeds a phase transformation from a hexagonal symmetry (H1 phase) to a monoclinic symmetry (M phase). This monoclinic phase of distorted O3 structure has a decreased lattice symmetry for *C2/m* compared to *R*$\bar{3}$*m*. The monoclinic phase can often be observed in layered cathodes because of lithium/vacancy ordering and/or collective Jahn-Teller (JT) distortion caused by the active cations such as Ni^3+^. While the lowering in symmetry from hexagonal to monoclinic generally occurs in a range of *x* ≈ 0.8−0.4 for the Li*_x_*TMO_2_ compositions. It further evolves into the H2 phase from M phase between *x* ≈ 0.40 and 0.36, and the H2 phase formed in *x* = 0.36 remains until *x* ≈ 0.26. Further, for the range of *x* ≈ 0.26−0.16, order H2-H3 phase transition occurs, which is usually found at ~4.2 V versus Li. Thus, the H3 phase, which has a substantially smaller *c* parameter compared to the H2 (Fig. S2c) [27], forms through a biphasic process despite the energetically costly and highly strained H3/H2 interface. It raises the question on the origin of the thermodynamic driving force for the phase separation. A similar two‐phase reaction, associated with a drastic decrease in the interlayer distance, is the phase transformation from O3 to H1‐3 observed in Li*_x_*CoO_2_ upon charging to ~4.6 V versus Li, which further transforms into an O1 structure with a higher extend of delithiation at a higher voltage range. Meanwhile, the H1-3 phase has never been reported for Ni-rich LTMOs, which implies that the H3 phase is unlikely to be an H1‐3 structure. Instead, a phase transition from H3 to O1 has been observed for Ni-rich LTMOs when charged to 4.2 V or 4.45 V, which suggests that the onset voltage for the occurrence of such phase transition should be lower than 4.2 V versus Li.

Importantly, the kinetics of this phase change can be very sluggish, as it is observed under a condition of extremely slow charging for the LNO samples with low amounts of antisite defects [39-41]. This observation is likely to be caused by the presence of Ni in the Li layer that hinders the complete delithiation process and therefore impede a change in the stacking sequence. Indeed, for Ni-rich LTMOs with antisite defects at a concentration greater than 7%, the H3 → O1 transition can no longer be observed [42]. Therefore, it is hypothesized that the presence of an appropriate amount of antisite defects in the material structure could be beneficial for Ni-rich LTMOs, as it can suppress the H3 →O1 transition.

- 1. **Complexity and evolution in LTMO’s electronic structure.**

The repeated energy storage and release in a “rocking chair” battery features a back-and-forth conversion process between electric energy and chemical energy. The total amount of energy produced/stored by discharging/charging a battery is determined by the change in the Gibbs free energy (Δ*G*) of the system [43]. Δ*G* can be measured by the maximum amount of work that can be performed during this energy conversion process, which is described by the classic Nernst Equation: Δ*G_S_* = *-nFE_S_*, where Δ*G_S_* is the change in the standard Gibbs free energy of the system, *n* is the number of the transferred electrons, *F* is the Faraday constant, and *E_S_* is the standard potential of the battery. That is to say, the total amount of charge transferred during the chemical reaction and the corresponding cell potential collectively determine the capacity of the battery. In LTMO-based batteries, these two key parameters are derived from the properties of the transition metal (TM) cations, e.g., Ni, Co, and Mn as well as the redox active anions, e.g., O. Therefore, it is vital to thoroughly characterize the electronic structures of these elements for a better understanding of the cathode properties and, subsequently, for improving the battery performance.

In practical electrochemical systems, the complexity in the redox reactions stems from several co-existing and intertwined redox centers. For example, due to the partial overlap between $t_{2g}$ band of Co and 2p band of O, both Co^3+^ and O^2-^ can be oxidized when more than 0.5 Li are extracted from the LCO lattice, leading to a capacity decay [28]. In pristine LiNi_1/3_Co_1/3_Mn_1/3_O_2_ (NMC-333) cathode the TMs’ initial oxidation states are Ni^2+^, Co^3+^, and Mn^4+^, respectively. The redox activities of Ni^2+^/Ni^4+^ and Co^3+^/Co^4+^ dominate the charge compensation below 0.65 delithiation, whereas the oxidation state of Mn^4+^ is mostly unchanged [44]. Oxygen, however, can impact the system throughout the entire potential window through TM-O hybridization. In particular, at a deeply delithiated state (above 0.65 Li-extraction), the oxygen anions could become redox active. It is also reported that the oxygen redox is related with its local TM species, which is rather complicated due to the participation of multiple elements [44]. This interplay has also been reported in Li_1-x_Ni_0.88_Co_0.1_Al_0.02_O_2_ (NCA) cathode [45]. It is suggested that the Co cation can compensate the charge in parallel with the Ni cation through t_2g_-e_g_ hopping. In Li-rich NMC materials, oxygen redox becomes an important contributor to its total capacity. In general, the lattice oxygen release/oxidation [46-48] or the formation of localized electron holes on the oxygen anions around Mn^4+^ and Li^+^ [49] are proposed as possible mechanisms of the oxygen redox process. The respective fractions of capacity contribution from Ni, Co, Mn, and O could dynamically evolve as the cell is repeatedly cycled [50]. This has been identified as one of the root causes for the voltage and capacity decay in Li-rich cathode materials.

To improve the performance of LTMO cathode, a number of different approaches have been carried out. Examples include doping and coating, structural design, and compositional optimization [51]. These approaches have demonstrated significant impacts on the LTMO’s electronic structures. As an example of the doping and coating approach, Titanium (Ti) was used to regulate the electronic structure on LCO surface to improve the stability of LCO at high voltage [52]. The unoccupied O 2p states above the Fermi level are largely suppressed on the Ti-rich surface, where the charge deficiency is decreased. The O anions around Ti cations lose less charge compared with those far from the Ti cations, inhibiting the irreversible oxygen redox [52]. The electronic conductivity of LCO can be improved by doping magnesium. By introducing ~5% Mg in LCO, the electronic conductivity is increased to ~0.5 S cm^−1^. That is because Mg^2+^ partly substitutes Co^3+^, leading to a decrease of Li vacancies in order to keep the charge neutrality [53]. In term of structural optimization, nanosized LCO has been considered as high-power cathode candidate, which benefits from its anisotropic surface and short Li-ion diffusion length [54]. In addition to nanosizing, the lattice facet engineering is also demonstrated to be effective. The valance state of Co deduced from surface to inner in (003) plane, whereas no apparent changing in the *ab*-plane [55]. The former can lead to surface effect along *c*-direction that benefits Li-ion diffusion. Compositional optimization is another popular approach that have shown great potential. In charged NMC cathode, the high valent Ni is active and can oxidized the electrolyte. This side reaction can lead to the loss of active materials as well as the increase of impedance. Sun *et al.* designed a full-gradient NMC material that is Mn-rich on the surface and Ni-rich in the core region [56]. The Mn’s 3d band is more inert and the Mn-rich surface can efficiently suppress the interphase side reactions and improve the cathode stability. Lin *et al.* demonstrated that the self-assemble effect in a spray pyrolysis process can also achieve a depth-dependent compositional heterogeneity with similar characteristics [19]. To suppress the voltage decay in Li-rich NMC materials, Sun *et al.* demonstrated a control of the local electronic structure through tailoring the chemical composition [57]. By increasing the Ni content in Li-rich NMC, the oxidation state of TMs, especially for Mn, can be increased. The TM 3d−O 2p band and non-bonding O 2p band shift toward lower energy, which increases the operating voltage and suppresses the voltage decay.

In general, the cathode materials’ electronic structures are critical to the battery performance because the redox behaviors are directly correlated with the energy density and stability of LTMO and their dynamic evolutions can further complicate the system. Synchrotron X-ray techniques have unique advantages in probing and resolving the electronic structures both in the bulk and at the surface of the LTMO cathode.

**Table S2.** Absorption edges of elements for LTMO materials.

|  | *K*-edge (eV) | *L_3_*-edge (eV) | *L_2_*-edge (eV) |
| --- | --- | --- | --- |
| Li | 54.7 | - | - |
| Ni | 8333 | 852.7 | 870 |
| Co | 7709 | 778.1 | 793.2 |
| Mn | 6539 | 638.7 | 649.9 |
| O | 543.1 | 18.2 | 18.2 |


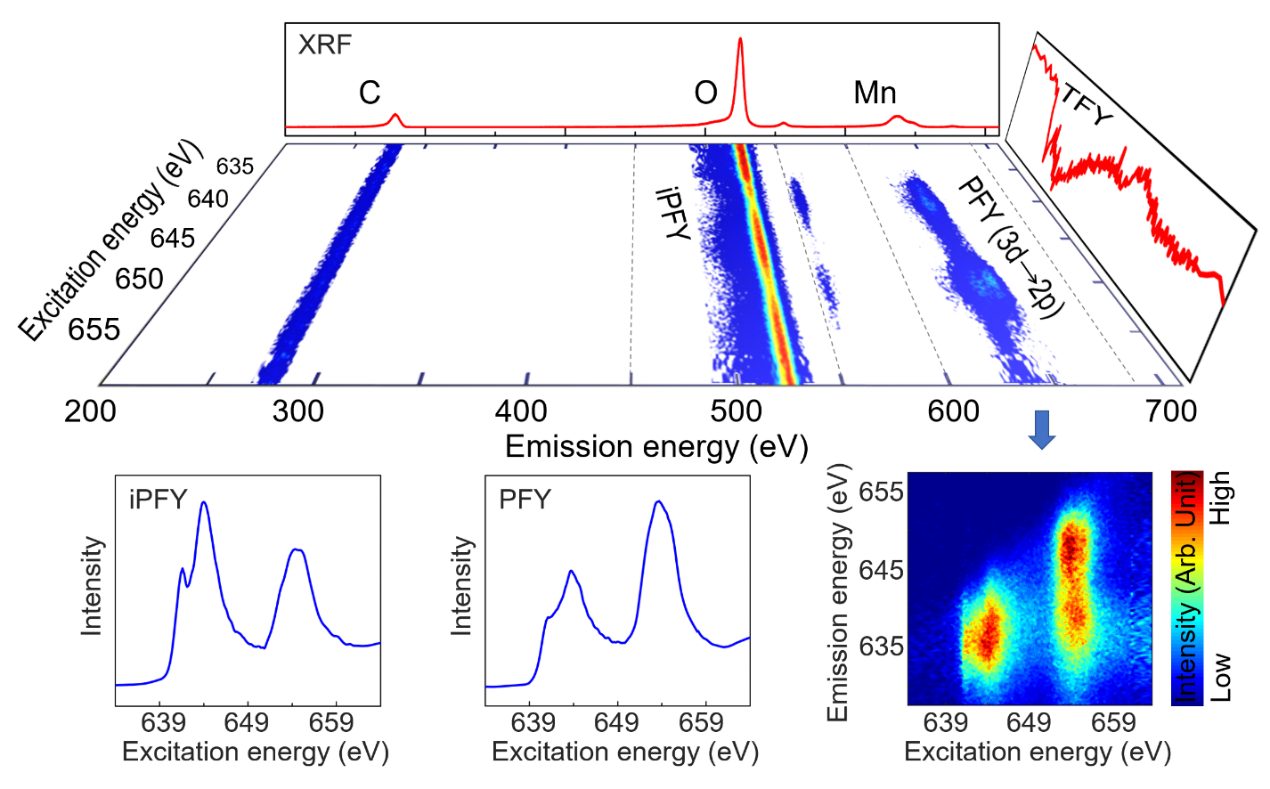


**Figure S3.** Mn RIXS map of the Li- and Mn-rich cathode at selected emission energy points from 200 to 700 eV. This two-dimensional map offers several different spectroscopic modalities including the XRF, TFY, PFY, iPFY, and RIXS, depending on which regions are selected and how the 2D maps are integrated. Specific to this case, the TFY signal shows severe distortion, while the PFY and iPFY spectra are free of this artifact.

- 1. **LTMO LIB’s hierarchical structure entails** **multi-scale degradation mechanisms.**

We start this section by discussing a cell-level synchrotron imaging study on LIBs under the thermal runaway conditions, which is a catastrophic effect that lies at the heart of the LIB’s safety concerns. As shown in Fig. S4(a-d), synchrotron-based high-frequency tomography and high-framerate radiography were employed to image the dynamic structural evolution of 18650-type commercial cells that were undergoing purposely provoked thermal runaway [58]. By using high-frequency synchrotron tomography, it was observed that the wound electrode layers of the cell with a built-in cylindrical support remained mostly intact before the thermal runaway (Fig. S4a), while noticeable electrode collapse occurred under a similar circumstance in a different cell that has no structural support (Fig. S4b). Although the synchrotron-base micro-tomography can reach to a data rate of ~1 tomogram per second or even slightly higher, it is temporal resolution is still far from sufficient when it comes to capturing the structural dynamics associated with the LIB’s thermal runaway. The authors turned to high-speed radiography with a high framerate of 1,250 frames per second (FPS), which largely benefit from the intense X-ray beam generated by a synchrotron facility. Key image frames presented in Fig. S4c-d demonstrate interesting morphological features that reveal the internal conditions of this burning LIBs. In the cell with structural support, the copper current collector melted and collected into globules during the thermal runaway, suggesting that the LIB’s internal temperature has reached above 1085 ℃, the melting point of copper, while the outer electrode layers remained unbroken, manifesting a huge temperature gradient. This was in part attributed to the ejection of the collapsed spiral-wound materials into the atmosphere, which effectively dissipated the heat. The authors further reported a distinct observation in the cell that has no structural support. Without the structural confinement, thermal runaway was not observed to propagate throughout the cell. The bulk of the intact contents were ejected soon after the initiation of the thermal runaway. This cell-level battery imaging with high temporal resolution provides a direct and dynamic visualization of the thermal runaway induced structural distortion, breakdown and material ejection, which is otherwise inaccessible. These observations can inform the thermal modelling effort to understand and, potentially, to mitigate the LIB failure with a thermal runaway.

**
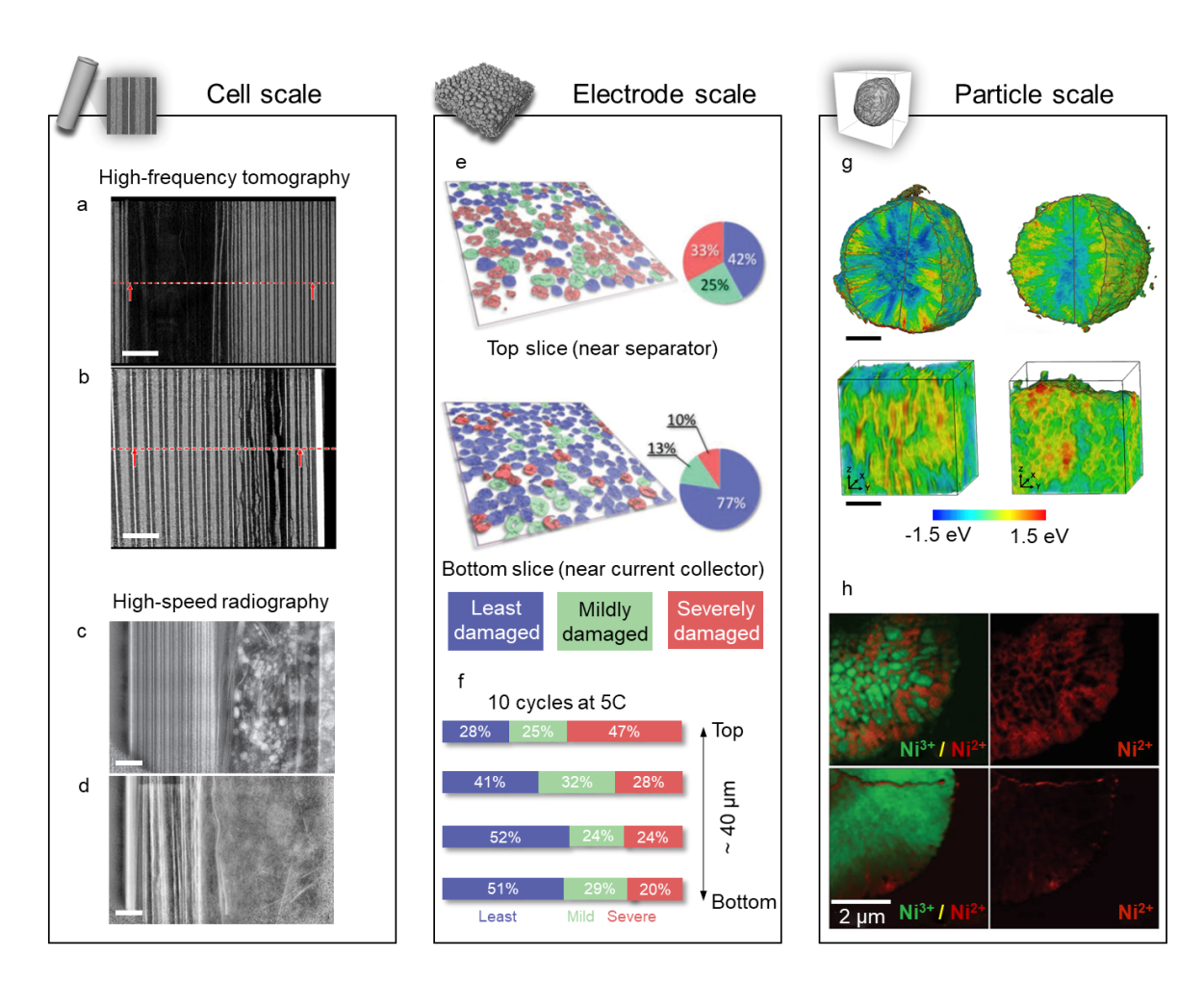
**

**Figure S4.** Synchrotron-based X-ray multi-scale imaging studies of LTMO cathode materials. (a-d) *Operando* high-speed tomography studies of 18650-type cell during thermal runaway, scale bar: 1 mm. Adapted with permission from [58], Copyright 2015, Springer Nature. (e) The degradation comparison of the top and the bottom layers in the LiNi_0.2_Mn_0.2_Co_0.2_O_2_ electrode. Adapted with permission from [59], Copyright 2019, WILEY‐VCH. (f) The fracturing profiles across the top to the bottom of the electrodes after 10 cycles at 5 C rate. Adapted with permission from [59], Copyright 2019, WILEY‐VCH. (g) Charge distributions in the first charged rod-NMC (left) and gravel-NMC (right) particles, scale bar: 3 μm. Adapted with permission from [60], Copyright 2020, Springer Nature. (h) Chemical phase maps (red and green represent Ni^2+^ and Ni^3+^, respectively) of cross sections of conventional (top) and “core-shell” structural (bottom) LiNi_0.9_Co_0.05_Mn_0.05_O_2_ particles in fully charged after 100 cycles. Adapted with permission from [61], Copyright 2021, WILEY‐VCH.

While the above discussed acute LIB failure through thermal runaway accentuates the need for rapid imaging capabilities at a synchrotron beamline, LIBs often experience chronic electrode degradations that feature a multi-scale and complicated electro-chemo-mechanical interplay. The root cause of the LIB degradation over repeated electrochemical cycling lies with the anisotropic lattice expansion and contraction in the active materials, which is accompanied by accumulation of mechanical stress, leading to structural disintegration. For an in-depth investigation of this effect, synchrotron microscopy techniques with high spatial resolution down to nanoscale become the go-to approach. In particular, the nano-resolution synchrotron X-ray tomography has been demonstrated as a powerful tool for detect the structural damage on the electrode to particle levels nondestructively. As a specific example, here we discuss a study on the nano-resolution imaging of polycrystalline NMC cathode by Yang *et al.* [59]. The polycrystalline NMC composite cathode is vulnerable to intergranular cracks along the grain boundaries of primary particles, which escalates the electrochemistry decay upon the cycling. While this cracking effect often occurs within individual secondary particles, at the electrode level, the degree of particle fracturing features a highly heterogeneous pattern after fast-charging protocols (as shown in Fig. S4e-f). A thorough quantification of these particles in a statistically significant manner is critical to avoiding any pitfall in the interpretation of the experimental results. The authors developed a machine learning method, which they reported in details in a follow-up publication by Jiang *et al.*[62], to conduct the particle identification and quantification automatically. A large number of NMC particles were segmented and labelled based on their respective degrees of fracturing. These results were then quantified for evaluating the depth-dependence in the electrode utilization at different states. In the electrode that was subjected to 10 fast cycles, it was observed that there are more severely damaged particles near the separator than near current collector, which is attributed to a strong cell polarization effect under fast charging conditions. Such a depth-dependent cracking pattern evolved towards a more homogeneous degradation pattern as more particles near the current collector participated in the electrochemical reactions during the later cycles. The authors fed this information into a systematic finite element modeling study and elucidated the underlying mechanism from a theoretical perspective [59]. In addition, thanks to the phase contrast capability in the synchrotron nanoprobe-based nano-tomography approach, noticeable detachment of the NMC particles’ from the carbon-binder domain (CBD) was also visualized in the severely damaged local regions [62], which could lead to partial deactivation of NMC particles and to a detriment to the capacity retention.

Despite that the active material particles could respectively exhibit different electrochemical states, they are the architectural building blocks for the electrode and a comprehensive investigation at the particle/sub-particle level can offer valuable insights from the fundamental perspective. At the particle level, the particle morphology has a significantly impact on its electrochemical performance, which, in turn, motivates very active particle engineering efforts in this field. Full-field synchrotron spectro-microscopy and spectro-tomography with ~30 nm spatial resolution has been successfully applied to the study of the battery particles and these efforts were termed the mesoscale battery science. In a recent study by Xu *et al.*, synchrotron spectro-tomography were employed to compare the 3D patterns of redox heterogeneity in NMC cathode particles with different primary grain arrangements (Fig. S4g) [60]. The particle with radially aligned rod grains exhibits a lower electrochemical polarization compared to the counterpart with randomly oriented gravel grains. With the chemical gradient analysis, it was confirmed that the radially aligned rod grains facilitate the Li-ion diffusion in a rather straight geometrical path with suppressed tortuosity, which, subsequently, improve the charge homogeneity.

As another example for the particle engineering efforts in this field, we refer to the construction of cathode particles with a “core-shell” characteristic that is featured by a compositional concentration gradient. This approach was pioneered by Sun *et al.* and they have demonstrated improvements in both specific capacity and cycling stability through this strategy [63]. For example, in the study of LiNi_0.9_Co_0.05_Mn_0.05_O_2_ doped with 1 mol% Al, the “core-shell” structure with aligned grains exhibits a distinct morphology comparing to the conventional structure with randomly oriented grains (Fig. S4h) [61]. The authors employed an energy-resolved transmission soft X-ray microscopy to detect the distribution of Ni valence state over the particle cross-section. Fig. S4h illustrates the morphology and composition modulated Ni valence state distribution over the particles that were harvested after 100 cycles. More Ni^2+^ was detected on the surface of the conventional NCMA particle as evidenced by red borders, suggesting that more severe surface reconstruction took place and produced NiO-like impurities that passivated the primary particles. In contrast, in the particle with core-shell compositional gradient, this damage effect was suppressed and most of the primary particles maintain the Ni^3+^ state.

**REFERENCES**

1. Norman D. X-ray absorption spectroscopy (EXAFS and XANES) at surfaces. *J Phys C: Solid State Phys* 1986; **19**: 3273-311.

2. de Groot F. High-Resolution X-ray Emission and X-ray Absorption Spectroscopy. *Chem Rev* 2001; **101**: 1779-808.

3. Adams F, Janssens K and Snigirev A. Microscopic X-ray fluorescence analysis and related methods with laboratory and synchrotron radiation sources. *J Anal At Spectrom* 1998; **13**: 319-31.

4. Weiland C, Rumaiz AK and Pianetta P *et al.* Recent applications of hard x-ray photoelectron spectroscopy. *J Vac Sci Technol A* 2016; **34**: 030801.

5. Kobayashi K. Hard X-ray photoemission spectroscopy. *Nucl Instrum Meth A* 2009; **601**: 32-47.

6. Lindau I, Pianetta P and Doniach S *et al.* X-ray photoemission spectroscopy. *Nature* 1974; **250**: 214-5.

7. Pérez CA, Radtke M and Sánchez HJ *et al.* Synchrotron radiation X-Ray fluorescence at the LNLS: beamline instrumentation and experiments. *X-Ray Spectrom* 1999; **28**: 320-6.

8. Kotani A and Shin S. Resonant inelastic x-ray scattering spectra for electrons in solids. *Rev Mod Phys* 2001; **73**: 203-46.

9. Ren Y and Zuo X. Synchrotron X-Ray and Neutron Diffraction, Total Scattering, and Small-Angle Scattering Techniques for Rechargeable Battery Research. *Small Methods* 2018; **2**: 1800064.

10. Kane PP. Inelastic scattering of X-rays and gamma rays. *Radiat Phys Chem* 2006; **75**: 2195-205.

11. Caldwell DO, Eisner AM and Elings VB *et al.* Measurements of Inelastic Compton Scattering. *Phys Rev Lett* 1974; **33**: 868-71.

12. Wu J, Yang Y and Yang W. Advances in soft X-ray RIXS for studying redox reaction states in batteries. *Dalton Trans* 2020; **49**: 13519-27.

13. Li J, Wang X and Zhao J *et al.* Porous lithium nickel cobalt manganese oxide hierarchical nanosheets as high rate capability cathodes for lithium ion batteries. *J Power Sources* 2016; **307**: 731-7.

14. Yang W and Devereaux TP. Anionic and cationic redox and interfaces in batteries: Advances from soft X-ray absorption spectroscopy to resonant inelastic scattering. *J Power Sources* 2018; **389**: 188-97.

15. Lin F, Liu Y and Yu X *et al.* Synchrotron X-ray Analytical Techniques for Studying Materials Electrochemistry in Rechargeable Batteries. *Chem Rev* 2017; **117**: 13123-86.

16. Li W, Li M and Hu Y*, et al.* Synchrotron-Based X-ray Absorption Fine Structures, X-ray Diffraction, and X-ray Microscopy Techniques Applied in the Study of Lithium Secondary Batteries. *Small Methods* 2018; **2**: 1700341.

17. Wei C, Xia S and Huang H *et al.* Mesoscale Battery Science: The Behavior of Electrode Particles Caught on a Multispectral X-ray Camera. *Accounts Chem Res* 2018; **51**: 2484-92.

18. Alvarado J, Wei C and Nordlund D *et al.* Thermal stress-induced charge and structure heterogeneity in emerging cathode materials. *Mater Today* 2020; **35**: 87-98.

19. Lin F, Nordlund D and Li Y *et al.* Metal segregation in hierarchically structured cathode materials for high-energy lithium batteries. *Nat Energy* 2016; **1**: 15004.

20. Tian C, Xu Y and Nordlund D *et al.* Charge Heterogeneity and Surface Chemistry in Polycrystalline Cathode Materials. *Joule* 2018; **2**: 464-77.

21. Xu Y, Hu E and Zhang K *et al.* In situ Visualization of State-of-Charge Heterogeneity within a LiCoO_2_ Particle that Evolves upon Cycling at Different Rates. *ACS Energy Lett* 2017; **2**: 1240-5.

22. Qian G, Zhang J and Chu S-Q *et al.* Understanding the Mesoscale Degradation in Nickel-Rich Cathode Materials through Machine-Learning-Revealed Strain–Redox Decoupling. *ACS Energy Lett* 2021; **6**: 687-93.

23. Mao Y, Wang X and Xia S *et al.* High-Voltage Charging-Induced Strain, Heterogeneity, and Micro-Cracks in Secondary Particles of a Nickel-Rich Layered Cathode Material. *Adv Funct Mater* 2019; **29**: 1900247.

24. Zhang J, Wang Q and Li S *et al.* Depth-dependent valence stratification driven by oxygen redox in lithium-rich layered oxide. *Nat Commun* 2020; **11**: 6342.

25. Delmas C, Fouassier C and Hagenmuller P. Structural classification and properties of the layered oxides. *Physica B & C*. 1980; **99**: 81-5.

26. Jarvis KA, Deng Z and Allard LF *et al.* Atomic Structure of a Lithium-Rich Layered Oxide Material for Lithium-Ion Batteries: Evidence of a Solid Solution. *Chem Mat* 2011; **23**: 3614-21.

27. Xu C, Reeves PJ and Jacquet Q *et al.* Phase Behavior during Electrochemical Cycling of Ni-Rich Cathode Materials for Li-Ion Batteries. *Adv Energy Mater* 2021; **11**: 2003404.

28. Liu W, Oh P and Liu X *et al.* Nickel-Rich Layered Lithium Transition-Metal Oxide for High-Energy Lithium-Ion Batteries. *Angew Chem Int Edit* 2015; **54**: 4440-57.

29. Zheng J, Teng G and Xin C *et al.* Role of Superexchange Interaction on Tuning of Ni/Li Disordering in Layered Li(Ni_x_Mn_y_Co_z_)O_2_. *J Phys Chem Lett* 2017; **8**: 5537-42.

30. Li HH, Yabuuchi N and Meng YS *et al.* Changes in the cation ordering of layered O3 Li_x_Ni_0.5_Mn_0.5_O_2_ during electrochemical cycling to high voltages: An electron diffraction study. *Chem Mat* 2007; **19**: 2551-65.

31. Jung S-K, Gwon H and Hong J *et al.* Understanding the Degradation Mechanisms of LiNi_0.5_Co_0.2_Mn_0.3_O_2_ Cathode Material in Lithium Ion Batteries. *Adv Energy Mater* 2014; **4**: 1300787.

32. Wu Y, Ma C and Yang J *et al.* Probing the initiation of voltage decay in Li-rich layered cathode materials at the atomic scale. *J Mater Chem A* 2015; **3**: 5385-91.

33. Eum D, Kim B and Kim SJ *et al.* Voltage decay and redox asymmetry mitigation by reversible cation migration in lithium-rich layered oxide electrodes. *Nat Mater* 2020; **19**: 419-427.

34. Zheng J, Xu P and Gu M *et al.* Structural and Chemical Evolution of Li- and Mn-Rich Layered Cathode Material. *Chem Mat* 2015; **27**: 1381-90.

35. Shunmugasundaram R, Arumugam RS and Dahn JR. A Study of Stacking Faults and Superlattice Ordering in Some Li-Rich Layered Transition Metal Oxide Positive Electrode Materials. *J Electrochem Soc* 2016; **163**: A1394-A400.

36. Breger J, Jiang M and Dupre N *et al.* High-resolution X-ray diffraction, DIFFaX, NMR and first principles study of disorder in the Li_2_MnO_3_-LiNi_1/2_Mn_1/2_O_2_ solid solution. *J Solid State Chem* 2005; **178**: 2575-85.

37. Meng YS, Ceder G and Grey CP *et al.* Cation ordering in layered O3 Li[Ni_x_Li_1/3-2x/3_Mn_2/3-x/3_]O_2_ (0<=x<=1/2) compounds. *Chem Mat* 2005; **17**: 2386-94.

38. Riou A, Lecerf A and Gerault Y *et al.* Structural study of Li_2_MnO_3_. *Mater Res Bull* 1992; **27**: 269-75.

39. Croguennec L, Pouillerie C and Mansour AN *et al.* Structural characterisation of the highly deintercalated Li_x_Ni_1.02_O_2_ phases (with x <= 0.30). *J Mater Chem* 2001; **11**: 131-41.

40. Croguennec L, Pouillerie C and Delmas C. NiO_2_ obtained by electrochemical lithium deintercalation from lithium nickelate: Structural modifications. *J Electrochem Soc* 2000; **147**: 1314-21.

41. Li H, Zhang N and Li J *et al.* Updating the Structure and Electrochemistry of Li_x_NiO_2_ for 0 <= x <= 1. *J Electrochem Soc* 2018; **165**: A2985-A93.

42. Croguennec L, Pouillerie C and Delmas C. Structural characterisation of new metastable NiO_2_ phases. *Solid State Ion* 2000; **135**: 259-66.

43. Schmidt-Rohr K. How Batteries Store and Release Energy: Explaining Basic Electrochemistry. *J Chem Educ* 2018; **95**: 1801-10.

44. Lee G-H, Wu J and Kim D *et al.* Reversible Anionic Redox Activities in Conventional LiNi_1/3_Co_1/3_Mn_1/3_O_2_ Cathodes. *Angew Chem Int Edit* 2020; **59**: 8681-8.

45. Lee W, Yun S and Li H *et al.* Anionic Redox Chemistry as a Clue for Understanding the Structural Behavior in Layered Cathode Materials. *Small* 2020; **16**: 1905875.

46. Zhao E, Li Q and Meng F *et al.* Stabilizing the Oxygen Lattice and Reversible Oxygen Redox Chemistry through Structural Dimensionality in Lithium-Rich Cathode Oxides. *Angew Chem Int Edit* 2019; **58**: 4323-7.

47. House RA, Marie J-J and Pérez-Osorio MA *et al.* The role of O_2_ in O-redox cathodes for Li-ion batteries. *Nat Energy* 2021; doi: 10.1038/s41560-021-00782-0

48. McCalla E, Abakumov AM and Saubanère M *et al.* Visualization of O-O peroxo-like dimers in high-capacity layered oxides for Li-ion batteries. *Science* 2015; **350**: 1516.

49. Luo K, Roberts MR and Hao R *et al.* Charge-compensation in 3d-transition-metal-oxide intercalation cathodes through the generation of localized electron holes on oxygen. *Nat Chem* 2016; **8**: 684-91.

50. Hu E, Yu X and Lin R *et al.* Evolution of redox couples in Li- and Mn-rich cathode materials and mitigation of voltage fade by reducing oxygen release. *Nat Energy* 2018; **3**: 690-8.

51. Liu L, Li M and Chu L *et al.* Layered ternary metal oxides: Performance degradation mechanisms as cathodes, and design strategies for high-performance batteries. *Prog Mater Sci* 2020; **111**: 100655.

52. Hong Y-S, Huang X and Wei C *et al.* Hierarchical Defect Engineering for LiCoO_2_ through Low-Solubility Trace Element Doping. *Chem* 2020; **6**: 2759-69.

53. Tukamoto H and West AR. Electronic Conductivity of LiCoO_2_ and Its Enhancement by Magnesium Doping. *J Electrochem Soc* 1997; **144**: 3164-8.

54. Okubo M, Hosono E and Kim J *et al.* Nanosize Effect on High-Rate Li-Ion Intercalation in LiCoO_2_ Electrode. *J Am Chem Soc* 2007; **129**: 7444-52.

55. Okubo M, Kim J and Kudo T *et al.* Anisotropic Surface Effect on Electronic Structures and Electrochemical Properties of LiCoO_2_. *J Phys Chem C* 2009; **113**: 15337-42.

56. Sun Y-K, Chen Z and Noh H-J *et al.* Nanostructured high-energy cathode materials for advanced lithium batteries. *Nat Mater* 2012; **11**: 942-7.

57. Sun G, Yu F-D and Que L-F *et al.* Local electronic structure modulation enhances operating voltage in Li-rich cathodes. *Nano Energy* 2019; **66**: 104102.

58. Finegan DP, Scheel M and Robinson JB *et al.* In-operando high-speed tomography of lithium-ion batteries during thermal runaway. *Nat Commun* 2015; **6**: 6924.

59. Yang Y, Xu R and Zhang K *et al.* Quantification of Heterogeneous Degradation in Li-Ion Batteries. *Adv Energy Mater* 2019; **9**: 1900674.

60. Xu Z, Jiang Z and Kuai C *et al.* Charge distribution guided by grain crystallographic orientations in polycrystalline battery materials. *Nat Commun* 2020; **11**: 83.

61. Park N-Y, Ryu H-H and Park G-T *et al.* Optimized Ni-Rich NCMA Cathode for Electric Vehicle Batteries. *Adv Energy Mater* 2021; **11**: 2003767.

62. Jiang Z, Li J and Yang Y *et al.* Machine-learning-revealed statistics of the particle-carbon/binder detachment in lithium-ion battery cathodes. *Nat Commun* 2020; **11**: 2310.

63. Sun Y-K, Myung S-T and Park B-C *et al.* High-energy cathode material for long-life and safe lithium batteries. *Nat Mater* 2009; **8**: 320-4.
